# Macrophages as determinants and regulators of systemic sclerosis-related interstitial lung disease

**DOI:** 10.1186/s12967-024-05403-4

**Published:** 2024-06-27

**Authors:** Shih-Ching Lee, Chen-Hao Huang, Yen-Jen Oyang, Hsuan-Cheng Huang, Hsueh-Fen Juan

**Affiliations:** 1 Graduate Institute of Biomedical Electronics and Bioinformatics, National Taiwan University, Taipei, 10617 Taiwan; 2 Division of Rheumatology, Allergy and Immunology, Department of Internal Medicine, Chang Gung Memorial Hospital, Taoyuan, 333 Taiwan; 3 Institute of Biomedical Informatics, National Yang Ming Chiao Tung University, Taipei, 11221 Taiwan; 4 Department of Life Science, National Taiwan University, Taipei, 106 Taiwan; 5 Center for Computational and Systems Biology, National Taiwan University, Taipei, 106 Taiwan

**Keywords:** Systemic sclerosis, Lung fibrosis, SSc-ILD, Macrophage, MAPK, IL6

## Abstract

**Background:**

Interstitial lung disease (ILD) is the primary cause of mortality in systemic sclerosis (SSc), an autoimmune disease characterized by tissue fibrosis. SSc-related ILD (SSc-ILD) occurs more frequently in females aged 30–55 years, whereas idiopathic pulmonary fibrosis (IPF) is more prevalent in males aged 60–75 years. SSc-ILD occurs earlier than IPF and progresses rapidly. FCN1, FABP4, and SPP1 macrophages are involved in the pathogenesis of lung fibrosis; SPP1 macrophages demonstrate upregulated expression in both SSc-ILD and IPF. To identify the differences between SSc-ILD and IPF using single-cell analysis, clarify their distinct pathogeneses, and propose directions for prevention and treatment.

**Methods:**

We performed single-cell RNA sequencing on NCBI Gene Expression Omnibus (GEO) databases GSE159354 and GSE212109, and analyzed lung tissue samples across healthy controls, IPF, and SSc-ILD. The primary measures were the filtered genes integrated with batch correction and annotated cell types for distinguishing patients with SSc-ILD from healthy controls. We proposed an SSc-ILD pathogenesis using cell–cell interaction inferences, and predicted transcription factors regulating target genes using SCENIC. Drug target prediction of the TF gene was performed using Drug Bank Online.

**Results:**

A subset of macrophages activates the MAPK signaling pathway under oxidative stress. Owing to the lack of inhibitory feedback from ANNEXIN and the autoimmune characteristics, this leads to an earlier onset of lung fibrosis compared to IPF. During initial lung injury, fibroblasts begin to activate the IL6 pathway under the influence of SPP1 alveolar macrophages, but IL6 appears unrelated to other inflammatory and immune cells. This may explain why tocilizumab (an anti-IL6-receptor antibody) only preserves lung function in patients with early SSc-ILD. Finally, we identified BCLAF1 and NFE2L2 as influencers of MAPK activation in macrophages. Metformin downregulates NFE2L2 and could serve as a repurposed drug candidate.

**Conclusions:**

SPP1 alveolar macrophages play a role in the profibrotic activity of IPF and SSc-ILD. However, SSc-ILD is influenced by autoimmunity and oxidative stress, leading to the continuous activation of MAPK in macrophages. This may result in an earlier onset of lung fibrosis than in IPF. Such differences could serve as potential research directions for early prevention and treatment.

**Supplementary Information:**

The online version contains supplementary material available at 10.1186/s12967-024-05403-4.

## Background

Systemic sclerosis (SSc) is an autoimmune disease characterized by tissue fibrosis, which can occur in the skin of the trunk, limb extremities, and visceral organs [1]. Lung fibrosis leads to interstitial lung disease (ILD), the primary cause of mortality in SSc [2], due to the lack of treatments that can stop or reverse the fibrotic process [3–6]. The prevalence of ILD in patients with SSc ranges from 25 to 90% depending on the SSc subtype and the criteria used to define ILD in different countries [7]. Systemic sclerosis-related interstitial lung disease (SSc-ILD) occurs primarily in females aged 30 and 55 years. Idiopathic pulmonary fibrosis (IPF) is more prevalent in males aged approximately 60–75 years. SSc-ILD occurs earlier than IPF and progresses more rapidly [1, 8].

Macrophages have been found to play a key role in pulmonary fibrosis by attracting immune cells and stimulating collagen overproduction [9–11]. Three types of macrophages are involved in the pathogenesis of lung fibrosis: FCN1, FABP4, and SPP1 [12, 13]. SPP1 macrophages bearing MERTK and LGMN are likely to promote profibrotic activity [14]. However, the SPP1 macrophage group showed upregulated expression in both SSc-ILD and IPF [10, 14].

Through single-cell analysis, we aimed to identify the differences between SSc-ILD and IPF to clarify their distinct pathogeneses and propose directions for prevention and treatment.

## Methods

### Single-cell RNA-sequencing dataset

Single-cell RNA-sequencing (scRNA-seq) data were obtained from the NCBI Gene Expression Omnibus (GEO) database (accession numbers GSE159354 and GSE212109). Any public, de-identified data available as open access was not subject to local institutional review board requirements or patient consent, as allowed under the Common Rule. Tissue samples from both datasets were derived from explanted lung tissues. The GSE159354 dataset included four IPF, three patients with SSc-ILD, and three healthy controls (HCs), while GSE212109 dataset included five patients with SSc-ILD and six HCs. ScRNA-seq was performed on the 10 × Genomics platform, followed by sequencing on the Illumina HiSeq 4000 and NovaSeq 6000 platforms. Sequencing reads were assembled and aligned against the GRCh38 human reference using Cell Ranger v3.1.0 (10 × Genomics).

### Data processing analysis and clustering with cell-type annotation

Expression count matrices were analyzed using the Seurat v4.3.0 R package [15]. Only cells with at least 500 features and no more than 20% of the total mitochondrial feature count were retained for the analysis. Normalization was performed using the log-normalization method. Approximately 3000 highly variable features were selected for sample integration using the mean/variance regression method. Sample integration and batch correction were performed using an anchor-based sample integration workflow for tissue samples [15]. The “Elbow plot” (Fig S1A) was performed using the elbow function, which is a ranking of principle components (PCs) based on the percentage of variance. An ‘elbow’ was observed around PC 39–40, suggesting that the majority of true signal was captured in the first 40 PCs. The FindClusters function was used to cluster the cells, with a resolution parameter of 0.8 returning the best results. Finally, clustering was performed using 40 principal component analyses with the integrated resolution of 0.8 of shared nearest neighbor (SNN) graph using the Louvain algorithm. A 2D visualization of the clusters was performed with uniform manifold approximation and projection (UMAP) using the RunUMAP function in Seurat. The differentially expressed genes (DEGs) in each cluster were identified using the FindAllMarkers function in Seurat. Cell types for each cluster were annotated using the CellMarker 2.0 database (http://yikedaxue.slwshop.cn/) [16].

### MCODE component and DEG analysis

Enrichment network visualization and MCODE component analysis were performed using Metascape [17] and Cytoscape [18]. Subsequently, the DEGs were analyzed using Gene Ontology (GO) and Kyoto Encyclopedia of Genes and Genomes (KEGG) pathway analyses with the ClusterProfiler 4.9.0 package [19].

### Trajectory analysis

Trajectory analysis (pseudotime) was performed using slingshot v1.4.0 [20], with the UMAP coordinates and cluster 2 designated as the input and starting point, respectively. Slingshot v1.4.0 is a tool that uses preexisting clusters to infer lineage hierarchies and aligns the cells in each cluster on a pseudo-time trajectory.

### Cell–cell interaction analysis

Cell–cell interaction was inferred using CellChat v1.6.1 [21]. Ligand–receptor interactions were analyzed using single-cell transcriptomic data across lung tissue samples from HCs, patients with IPF, and patients with SSc-ILD.

### Gene regulatory network

The transcription factor (TF) gene regulatory network was constructed and TFs that may be responsible for the activation of target genes were identified using single-cell regulatory network inference and clustering (SCENIC) v1.3.1 [22]. In SCENIC, area under the curve (AUC) values were normalized to the Normalized Enrichment Score (NES). A high NES indicated a motif that recovered a large proportion of the input genes within the top ranking. The default cutoff was 3.0, which corresponds to a False Discovery Rate (FDR) of 3–9%. Significant motifs were associated with TFs using annotation databases for *Homo sapiens* [22].

### Drug-TF prediction and drug2cell

Drug target prediction of the TF gene was performed using the Drug Bank Online (https://go.drugbank.com/) [23]. For drug repurposing analysis, the drug scores in each single cell were calculated based on the target gene expression levels using the drug2cell package in Python (http://github.com/Teichlab/drug2cell) [24]. All drugs tested and selected had the statistically highest score in the cluster of macrophages according to the Wilcoxon sum test, and *p* values were adjusted using the Benjamini–Hochberg method [24]. Drug and target gene information was obtained from ChEMBL [25].

## Results

### ILD sample analysis

**Fig. 1:**
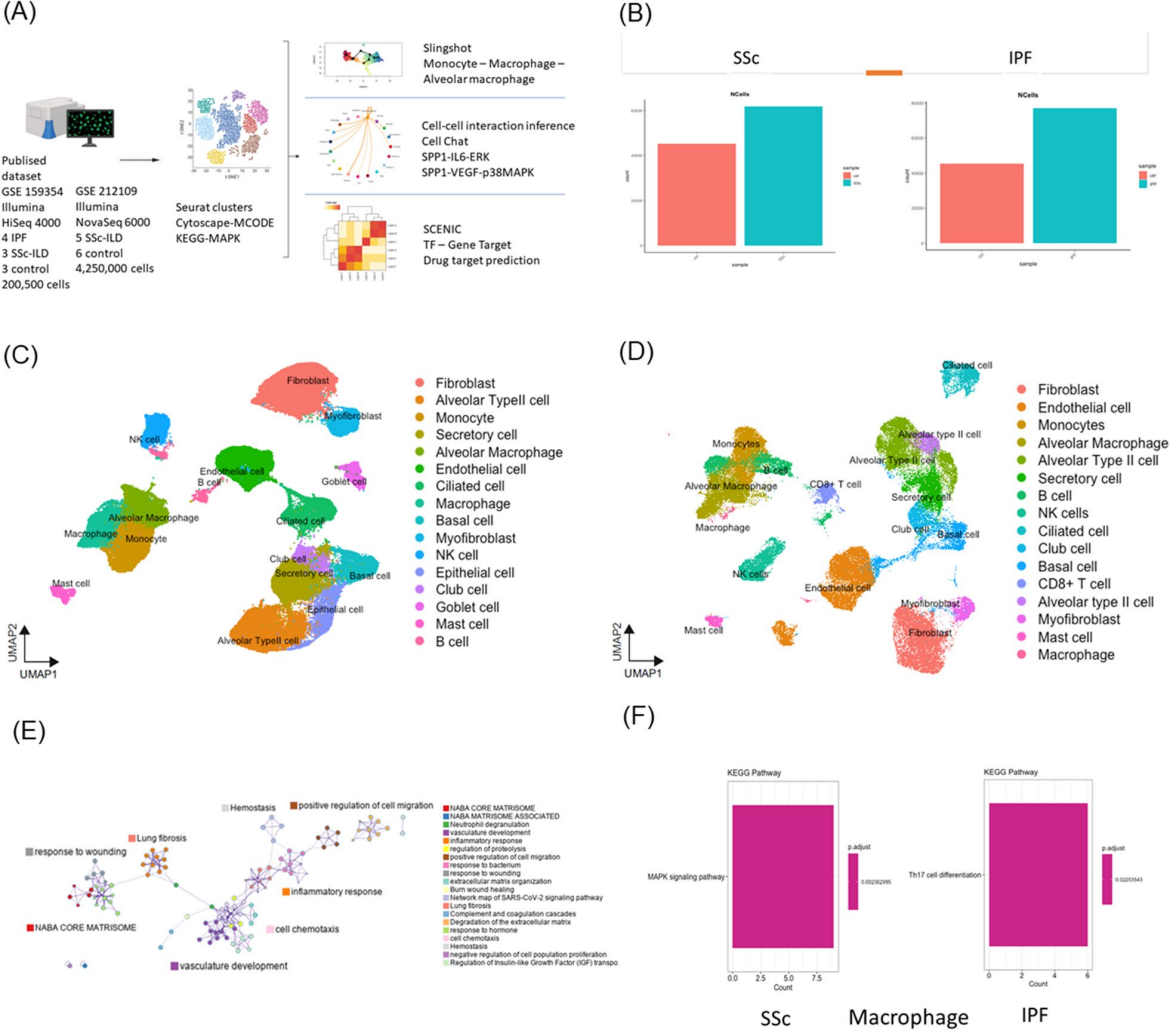
Single-cell RNA-sequencing analysis of lung samples from sysetmic sclerosis (SSc), idiopathic pulmonary fibrosis (IPF) and healthy controls (HCs). **A** Overview of study design and analysis. **B**–**F** Overview of the scRNAseq landscape of GSE159354. Markers were used to identify the clusters and differences among the SSc-ILD, IPF, and HC groups. **B** The analyzed cell counts of the SSc-ILD, IPF, and HC groups. UMAP of samples from the (**C**) SSc-ILD and HC group and (**D**) IPF and HC group. **E** Enriched ontology clusters of all samples. **F** Bar plots showing the KEGG pathways of macrophages in SSc-ILD and IPF. Illustrations in **A** were created using BioRender (http://biorender.com)

We analyzed lung samples from four patients with IPF, three patients with SSc-ILD patients, and three HCs from GSE159354 using scRNA-seq analysis (Fig. 1A). The filtered genes were integrated with batch corrections using the Seurat software. We conducted a bioinformatics analysis of samples in two groups of patients with SSc-ILD or IPF with HCs. In total, approximately 200,500 cells were analyzed (Fig. 1B). A total of 19 and 23 clusters were identified in the SSc-ILD with HCs (Fig. 1C and Fig S1B, C) and IPF with HCs groups (Fig. 1D), respectively. Based on the marker genes that were used to annotate and identify the clusters, the cell clusters were similar in both groups (Fig. 1C and D).

### Lung macrophages execute MAPK signaling pathway specific for SSc-ILD

In the enriched ontology clusters, we observed two cluster groups associated with lung fibrosis: the inflammatory process and complement cascade, and the wound healing process (Fig. 1E). Because of the similar clustering patterns and GO exhibited by these two sets of samples (SSc-ILD and IPF) (Fig S1D, E), we conducted an MCODE component analysis (Fig S1F). We identified the mitogen-activated kinase phosphatase (MAPK) signaling pathway in SSc-ILD, but not in IPF (Fig S1F). Therefore, we aimed to determine which group of cells was executing MAPK. KEGG analysis on fibroblasts, epithelial cells, monocytes, alveolar macrophages, and macrophages revealed that macrophages executed MAPK (Fig S1G–J, Fig. 1F); in contrast, the same group of macrophages in IPF underwent Th17 cell differentiation (Fig. 1F).

### Monocyte-derived lung macrophages

**Fig. 2:**
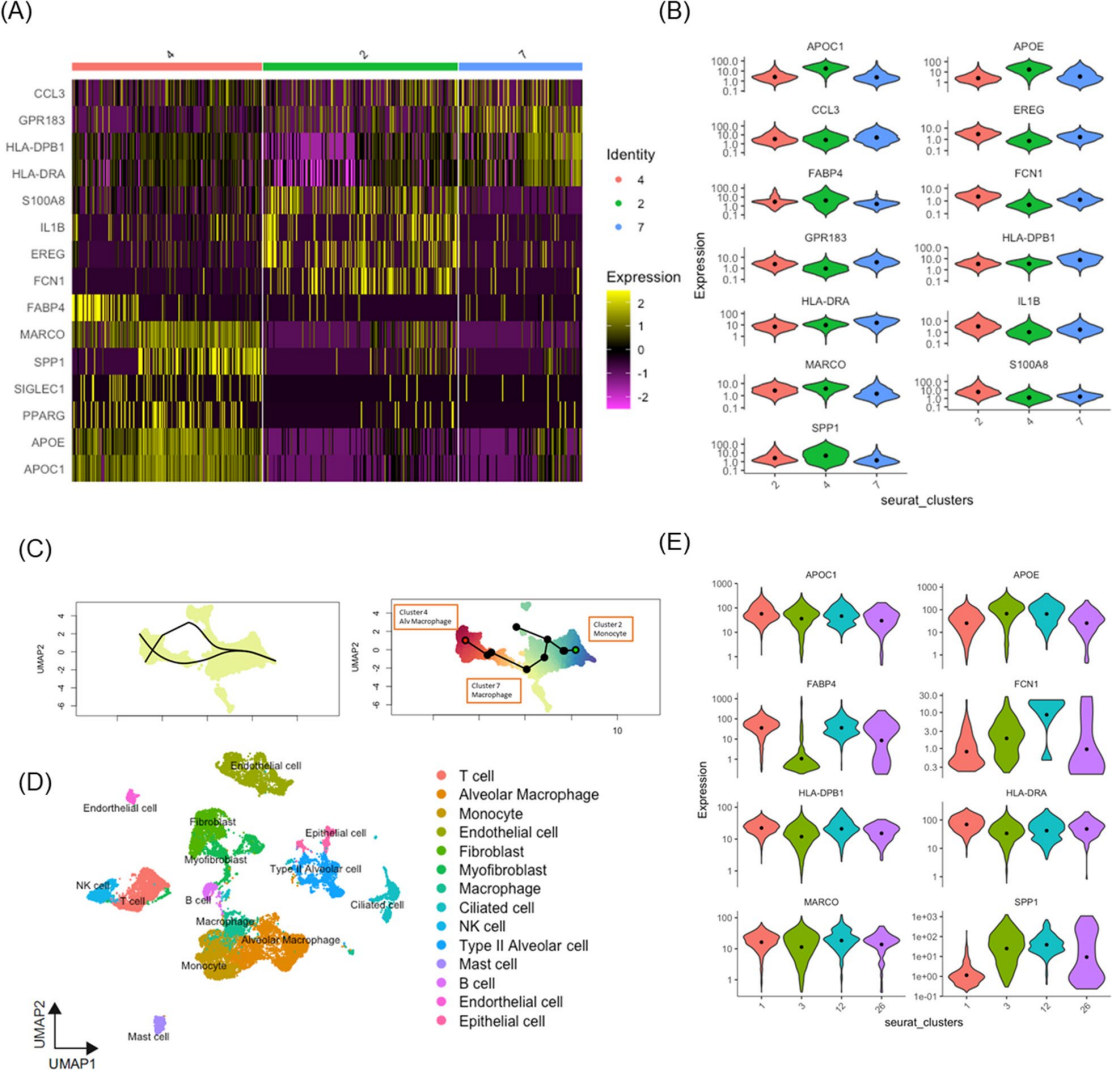
Single-cell RNA-sequencing analysis of monocyte/macrophage from systemic sclerosis (SSc). **A**–**C** Overview of the scRNAseq landscape of GSE159354. **A** Differential expression of key gene heatmap across cluster 4-alveolar macrophage, cluster 2-monocyte, and cluster 7-macrophage of SSc-ILD. **B** Violin plots showing the differential expression of key genes across cluster 2-monocyte, cluster 4-alveolar macrophage, and cluster 7-macrophage of SSc-ILD. **C** Suggested trajectory from monocytes, macrophage, and alveolar macrophages of SSc-ILD on the 2D map. **D**, **E** Overview of the scRNAseq landscape of GSE212109. Markers were used to identify the clusters and differences between SSc-ILD and HC. **D** UMAP of samples from the SSc-ILD and HC group. **E** Violin plots showing the differential expression of key genes across clusters 1, 3, 12, and 26

In the three Seurat clusters representing monocytes/macrophages of clusters 2, 4, and 7, the differentially expressed genes (DEGs) included FCN1, S100A8, and IL1B in cluster 2; APOC1, MARCO, FABP4, and SPP1 in cluster 4; and HLA-DPB1, GPR183, and CCL3 in cluster 7 (Fig. 2A). According to the CellMarker 2.0 database annotation [16], clusters 2, 4, and 7 corresponded to monocytes, alveolar macrophages, and macrophages, respectively (Fig. 2B). To determine the origin of the macrophages within these clusters, we performed slingshot [20] to distinguish the similarities between these three cell types. Starting with monocytes, we found that, to reach alveolar macrophages, one must first pass through the macrophages. There were distinct differentiation pathways from monocytes to alveolar macrophages and macrophages (Fig S1K). Thus, macrophages are more closely related to monocytes, whereas alveolar macrophages likely arrive in the lung tissue earlier and undergo more differentiation, resulting in notable differences from monocytes. Consequently, we concluded that macrophages were more likely to differentiate from monocytes (Fig. 2C).

### The alveolar macrophage subpopulation in SSc-ILD

Using the published dataset GSE 212109, we analyzed lung samples from five patients with SSc-ILD and six HCs (Fig. 1A). In the scRNA-seq analysis, 27 clusters were identified (Fig. 2D). Based on the marker genes used for annotating clusters and assigning identifications, clusters 2 and 7 corresponded to monocytes, cluster 8 to macrophages; and clusters 1, 3, 12, and 26 to alveolar macrophages (Fig S1L). In the alveolar macrophage subset, cluster 1 consisted of cells expressing markers for both FABP4 and MARCO, clusters 3 and 26 represented cells expressing SPP1, and cluster 12 comprised cells expressing both FABP4 and SPP1 (Fig. 2E). This observation aligns with the published dataset GSE159354, as we identified two groups of cluster 4-alveolar macrophage, one with the key genes FABP4 and MARCO, and the other with MARCO and SPP1 (Fig. 2A).

### Cell–cell interaction inference (ligand–receptor interaction)

**Fig. 3:**
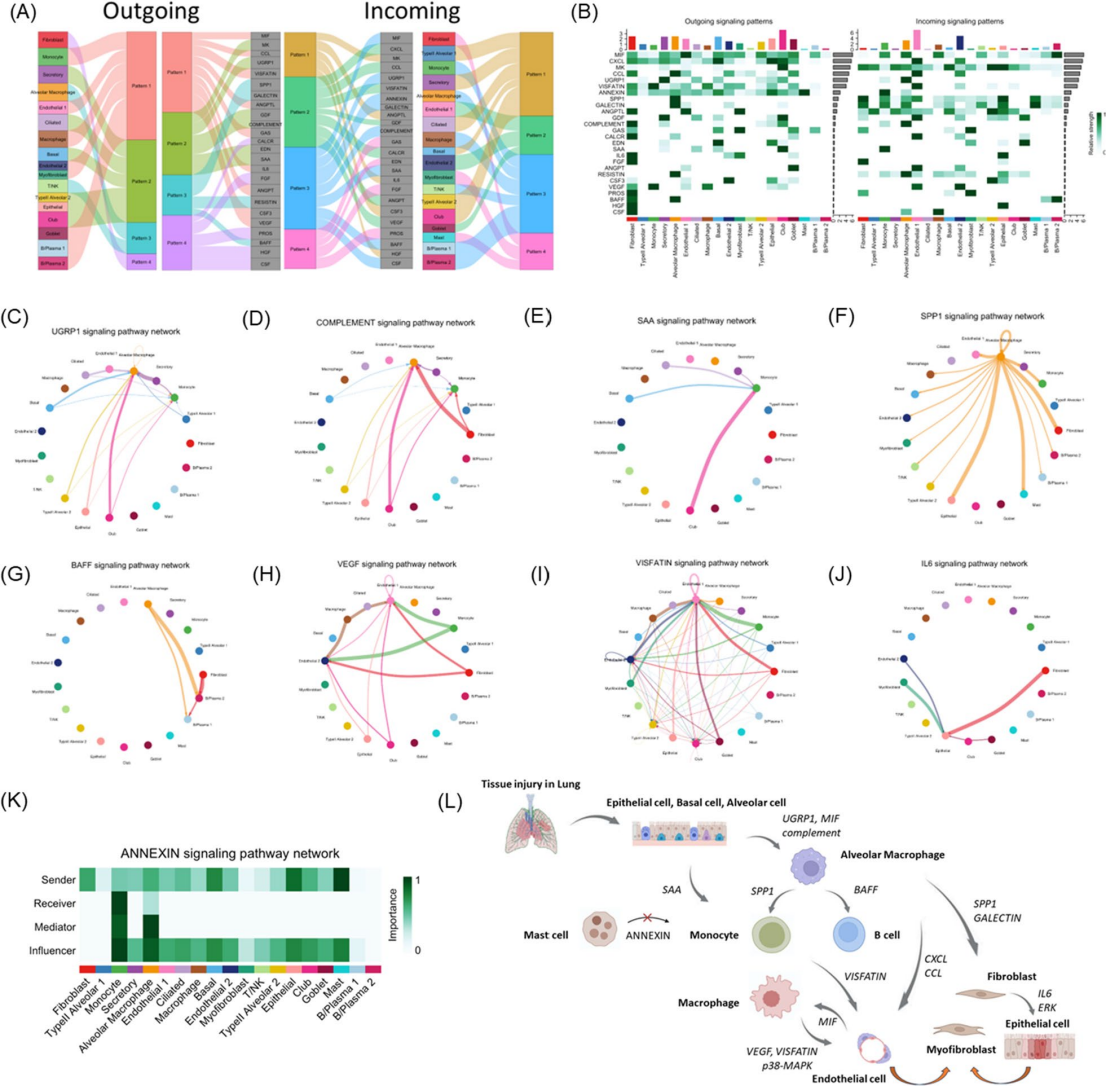
Functional differences between cell cluster interactions for candidate signaling pathways. **A** River plots of outgoing and incoming signal between cell clusters in SSc. **B** Heatmap highlighting the differential cell interaction strengths of outgoing and incoming signals. **C**–**J** Plots showing cell–cell interaction and strength for specific pathways, including (**C**) UGPR1, (**D**) COMPLEMENT, (**E**) SAA, (**F**) SPP1, (**G**) BAFF, (**H**) VEGF, (**I**) VISFATIN, and (**J**) IL6. **K** Heatmap illustrating cell–cell interaction and strength of ANNEXIN signaling pathway. **L** Graphical abstract of the lung single-cell interaction map of SSc-ILD. Illustrations were created using BioRender (http://biorender.com)

Given the differences in gene expression, our focus shifted to understanding how intercellular interactions may contribute to SSc-ILD and potentially lead to earlier disease onset compared to IPF [8]. Through functional differences between cell cluster interactions for candidate signaling pathways, we initially observed that type II alveolar cells, epithelial cells, secretory cells, and goblet cells tended to produce UGPR1, SAA, and complement signaling pathways upon injury (Fig. 3A, B, Fig S2A, B). UGPR1 activates alveolar macrophages bearing MARCO (Fig. 3C) [26], whereas the complement signaling pathway influences alveolar macrophages to drive additional immune and repair responses (Fig. 3D). In contrast, SAA acts on monocytes and triggers subsequent inflammatory reactions (Fig. 3E) [27].

Subsequently, alveolar macrophages produced SPP1 (Fig. 3F, Fig S2C) and BAFF (Fig. 3G). SPP1 not only acts on fibroblasts and epithelial cells for immediate repair (Fig. 3F), but also targets monocytes, further initiating an alternative repair pathway through the VEGF (Fig. 3H) and VISFATIN signaling pathway (Fig. 3I). Within monocytes, a distinct subset of macrophages executes this signaling pathway by specifically interacting with endothelial cells (Fig S2D, E). BAFF interacts with B cells to produce autoantibodies that are believed to be related to the autoimmune response [28].

We also observed that IL6, generally considered an inflammatory cytokine, was not produced by typical immune or inflammatory cells. Instead, it is generated by fibroblasts, myofibroblasts, and endothelial cells, and it acts on epithelial cells to facilitate repair processes (Fig. 3J). We also found that many cells, especially mast cells, produce ANNEXIN, which primarily acts on monocytes and alveolar macrophages, without affecting macrophages (Fig. 3K). ANNEXIN inhibits inflammatory responses via formyl peptide receptors (FPRs) [29]. However, macrophages lacked the annexin-FPR axis signaling pathway (Fig S2F). When macrophages are not inhibited by ANNEXIN, this may be the reason for the continued action of VEGF and VISFATIN.

We found that mast cells play a pivotal role in the feedback inhibition of monocytes. In previous studies, monocytes were found to possess two annexin receptors, FPR1 and FPR2. FPR1 primarily exhibits anti-inflammatory effects, while FPR2 aids the differentiation of monocytes into macrophages for efferocytosis [30]. However, in SSc-ILD, once monocytes differentiate into macrophages, both FPR1 and FPR2 disappeared (Fig S2F), consequently eliminating feedback inhibition from mast cells on macrophages. In contrast, monocytes in IPF possess only FPR1, and after differentiation into macrophages, the remaining FPR1 appears. Therefore, in IPF, feedback inhibition by mast cells persisted among monocytes, alveolar macrophages, and macrophages (Fig S2G).

### The biological pathway and pathogenesis of SSc-ILD

The biological pathway of SSc-ILD is outlined in Fig. 3L. When frontline cells in the lung tissue such as type II alveolar, epithelial, secretory, and goblet cells are exposed to external or internal damage, they initially send UGPR1 signals to alveolar macrophages. Consequently, alveolar macrophages activate fibroblast and other cells, such as myofibroblasts and endothelial cells, through SPP1. Endothelial cells can act as a source of myofibroblasts through the endothelial-to-myofibroblast transition (EndMT) and concomitant microvascular rarefaction [31]. Subsequently, through the IL6, ERK, and pI3k-Akt pathways [32], they drive cells such as epithelial cells and fibroblasts to continue repairing the injured tissue. The repair process contributes to lung fibrosis.

Additionally, SPP1 produced by alveolar macrophages drives monocytes, and within the recruited monocytes, some differentiate into specific macrophages activating the VEGF and VISFATIN signaling pathways due to the hypoxic characteristics of SSc, further driving the downstream p38-MAPK pathway [28, 33]. This process contributes to the development of lung fibrosis.

While there are mechanisms in place to inhibit alveolar macrophages and monocytes, especially by mast cells through ANNEXIN to suppress inflammation [34], this feedback mechanism does not act on macrophages. As a result, inflammation caused by oxidative stress cannot be inhibited, leading to a vicious cycle and an early accelerated onset of inflammation-induced lung fibrosis. Alveolar macrophages also drive B cells to produce autoantibodies through BAFF, which may continuously affect autoimmunity [35].

### p38-MAPK and JUN of the MAPK signaling pathway in lung macrophage

**Fig. 4:**
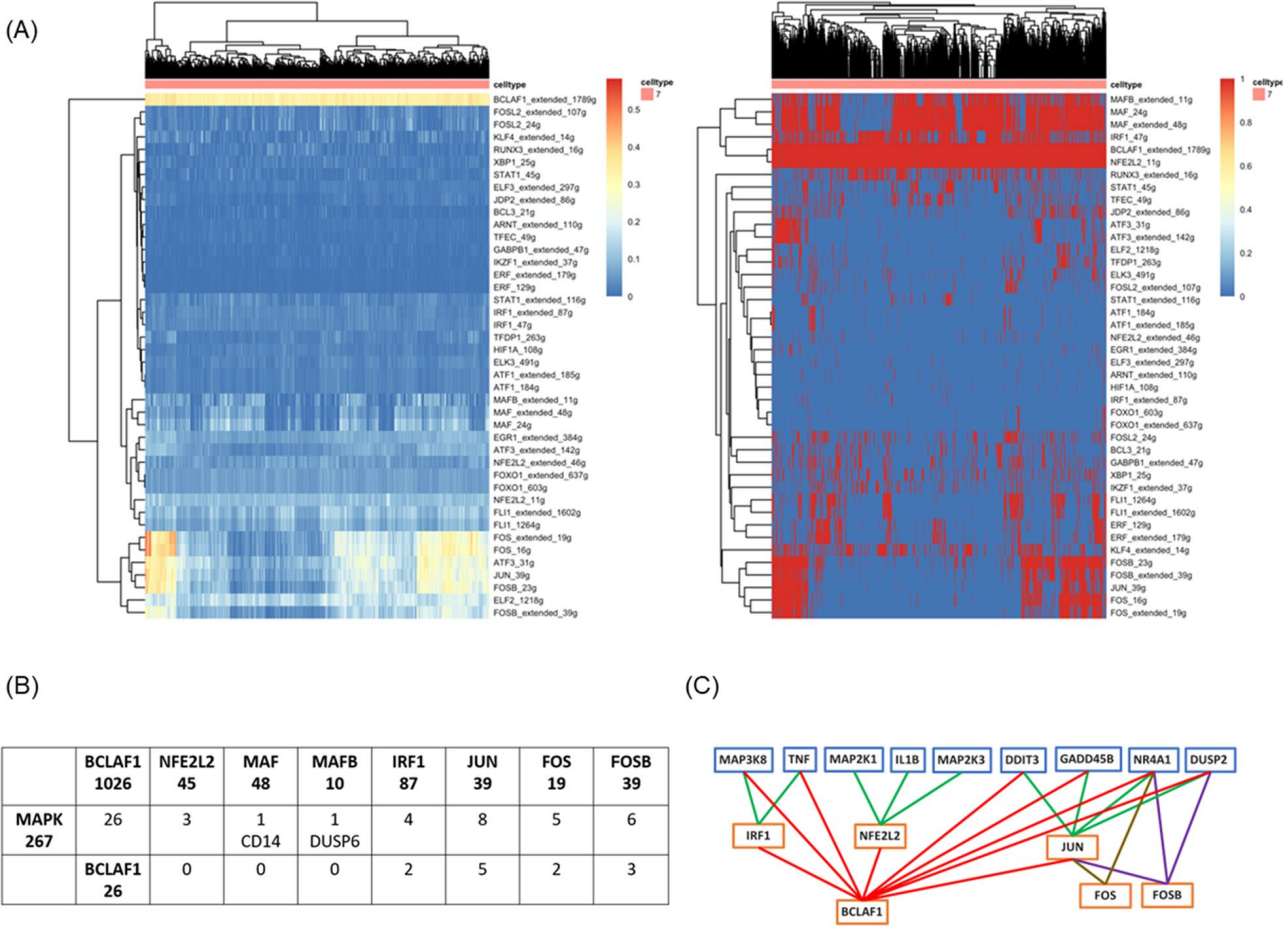
Regulons (transcription factor and downstream target genes) identified using SCENIC. **A** Heatmap of upregulated regulons of macrophages in samples of SSc-ILD. The AUC and binary matrix. **B** The upregulated regulons of macrophages overlapping with the MAPK pathway. **C** Networks of transcription factors and downstream target genes

We observed that this specific subset of macrophages activating the MAPK signaling pathway led to an earlier onset of SSc-ILD. SCENIC was used to predict TFs and putative target genes (Fig S3). We found two regulon groups: the first group included BCLAF1, IRF1, and NFE2L2, and the second group included JUN, FOS, and FOSB (Fig. 4A). From these two regulon groups, we identified that the target genes of each TF belonging to the MAPK signaling pathway (Fig. 4B). Within the TF and target gene networks, BCLAF1 regulates IRF1, NFE2L2, and JUN. Additionally, JUN is regulated by FOS and FOSB, which influence downstream genes (Fig. 4C). These two groups of genes were related to the p38-MAPK and JUN pathways (Table S1).

### Drug targeting prediction of transcription factors

Macrophage TFs are important targets of the MAPK signaling pathway. We mapped drugs targeting these TFs using the DrugBank database (https://go.drugbank.com/) (Table S2) [23]. Among the potential drugs, we selected those with downregulated effects on TFs and excluded those with controversial outcomes or those causing upregulated effects on other TFs. Additionally, we narrowed down the selection to those with an adjusted *p*-value < 0.05 (Table 1). Finally, we found that metformin downregulated NFE2L2. Metformin is a biguanide drug that is used to treat type 2 diabetes. It also has advantages in other diseases, including cancers and liver and renal diseases [36]. Previous reports suggest that metformin can inhibit the phosphorylation of Raf and ERK in a dose-dependent manner, thereby further suppressing NFE2L2 expression [37, 38].

**Table 1:** The drug scores of down regulated effect drugs to specific transcription factors

TF	CHEMBL Drug	Intersection	Gene group	Markers	Universe	p value	p value adj
NFE2L2	CHEMBL1703 METFORMIN HYDROCHLORIDE	50	51	8297	28,583	4.55E-26	5.20E-23
BCLAF1	CHEMBL38 TRETINOIN	7	12	8297	28,583	0.03237	0.72516
BCLAF1	CHEMBL98 VORINOSTAT	4	9	8297	28,583	0.24839	0.87621
BCLAF1	CHEMBL408513 BELINOSTAT	5	11	8297	28,583	0.18936	0.87621
JUN	CHEMBL313972 MASOPROCOL	11	21	8297	28,583	0.02062	0.61638
FOS FOSB	CHEMBL964 DISULFIRAM	12	26	8297	28,583	0.04777	0.78579

*TF* transcription factor

## Discussion

Lung fibrosis leads to ILD, which is the leading cause of death in patients with SSc [3]. In most individuals, SSc begins with Raynaud phenomenon (RP) [1]. RP is present for several years before the appearance of fibrosis. In the first 3 years after RP onset, approximately one-third of patients acquire a diffusion capacity of carbon monoxide (DLCO) < 50% of the predicted value [39]. Whatever the primary trigger, at the cellular level, a slight increase in reactive oxygen species (ROS) generates mild oxidative stress early in the disease, coinciding with endothelial-cell abnormalities and initial perivascular inflammation [28]. These mild abnormalities are responsible for subtle vascular dysfunction that does not manifest clinically [1].

A previous review article mentioned that systemic sclerosis in the development of ILD occurs earlier than IPF and progresses rapidly [8]. When lung injury occurs, frontline cells and tissues transmit UGPR1 signals to MARCO-bearing alveolar macrophages [26]. In adult lungs, at least two ontologically distinct populations of alveolar macrophages are present. Tissue-resident alveolar macrophages develop outside the bone marrow, differentiate into alveolar macrophages shortly after birth, self-renew, and persist throughout their lifespan. Monocyte-derived alveolar macrophages, develop from circulating monocytes and are recruited to the lungs during injury. Alveolar macrophages are critical resident cells in the alveolus and are important for both lung homeostasis and response to injury [40]. The key tissue-resident alveolar macrophage genes include FABP4, MARCO, and PPARγ [13], and key genes of monocyte-derived alveolar macrophages (interstitial macrophages) are SPP1 and LGMN. Another group of recruited macrophages contains the key genes FCN1 and S100A8 [12]. This observation aligns with our results, as we identified two groups of cluster 4-alveolar macrophages, one with FABP4 and MARCO, and the other with MARCO and SPP1. Furthermore, within cluster 2-monocyte, we observed FCN1 and S100A8 (Fig. 2A).

When alveolar macrophages receive UGPR1, they release SPP1 into the fibroblasts. Fibroblasts further activates the IL6 signaling pathway and ERK and pI3K-AKT downstream to promote the proliferation of myofibroblasts and epithelial cells for wound repair [32]. Fibroblasts are major contributors to and regulators of inflammation and dominant producers of IL6 in inflammatory diseases like rheumatoid arthritis and systemic sclerosis [41, 42]. A previous study revealed that ERK inhibition prevents the progression of lung fibrosis [43]. In particular, the IL6 pathway is not associated with immune or inflammatory cells, suggesting that this process occurs during the early stages of injury. This may explain why tocilizumab (an anti-IL6-receptor antibody) only preserves lung function over 48 weeks in patients with early SSc-ILD [5].

SPP1, produced by alveolar macrophages, also stimulates monocytes and a specific subset of macrophages in response to oxidative stress and ROS-mediated signaling. A previous study reported that the SPP1 can stimulate macrophages to activate adjacent endothelial cells, pericytes, and smooth muscle cells [44]. Through VEGF and VISFATIN, they act on endothelial cells promoting proliferate, migrate, and performing angiogenesis [31]. The ROS signaling pathway initially responds to PDGF triggers, after which the circuitry becomes a vicious cycle [28, 45]. Anti-PDGFR autoantibodies in systemic sclerosis possess biological activities and may contribute as pathogenic factors in tissue damage [46]. Most systemic sclerosis patients have circulating antinuclear autoantibodies in their blood, the most common being anti-topoisomerase I (ATA) and anti-centromere (CENP) antibodies. Although these antibodies can serve as serological hallmarks for early and precise diagnosis, their pathogenic features remain unknown. Several other autoantibodies, such as anti-fibroblasts, anti-endothelial cells, anti-fibrillin-1, anti-endothelin type A receptor, and anti-angiotensin II type 1 receptors have been identified, but their detection varies significantly, and their roles in SSc pathogenesis remain uncertain [28].

In TF prediction, we found two regulon groups: the BCLAF1/NFE2L2 complex and the FOS/JUN complex. In previous studies, the FOS/JUN complex was recognized as a pioneering transcription factor, serving as an enhanced selector that modulates DNA accessibility in fibroblasts, leading to the subsequent development of pulmonary fibrosis [47]. However, in our analysis, this group of macrophages did not directly affect fibroblasts (Fig S2E).

Through this process, most cells, particularly mast cells, secrete ANNEXIN to inhibit feedback on monocytes and alveolar macrophages. However, it did not affect the macrophages (Fig. 3K). Macrophages are the primary executors of VEGF, VISFATIN, and the subsequent p38-MAPK signaling pathway in the immune response. In a study by Matsuda et al. [48], elevated p38-MAPK signaling in the lungs was correlated with an increased severity of bleomycin-induced pulmonary fibrosis. Consequently, in the pathogenesis of SSc-ILD, this portion remains unimpeded by feedback inhibition, possibly leading to a continuous cycle of inflammation and repair, which ultimately results in lung fibrosis.

Hypoxia is the critical factor that induces transcription of the hypoxia inducible factor-1α (HIF-1α) accumulation to promote angiogenesis. When macrophage responds to hypoxia and ROS signaling, TFs such as BCLAF1, NFE2L2 (Nrf2), and HIF-1α become involved. BCLAF1 promotes HIF-1α transcription, leading to increased transcription of downstream targets, including VEGF [49]. As NFE2L2 is required for HIF-1α stabilization, it initially downregulates in response to hypoxia and later increases during reoxygenation, leading to antioxidant and cytoprotective gene expression [50].

Our study has several limitations. First, all tissue samples in these datasets were from lung transplantation patients; therefore, we could not observe cellular changes and pathogenesis at the early stages of SSc-ILD. Further cohort studies of patients with initial SSc-ILD are required to conduct such analyses. Additionally, the effects of BCLAF1 and NFE2L2 on HIF-1α during hypoxia have only been confirmed in liver and kidney tissues, with no reports in the lung tissues. Whether the genes related to MAPK observed in macrophages are involved in lung fibrosis requires further confirmation. Third, many reports suggest that the early onset of SSc-ILD may be influenced by the ROS signaling pathway affected by autoantibodies [28, 35]. However, the expression of anti-PDGFR antibodies among patients with SSc is quite variable. Therefore, whether there are other autoantibodies that are the primary cause of autoimmune pathology should be investigated.

## Conclusions

We found that during the initial lung injury in SSc-ILD, fibroblasts begin to activate the IL6 pathway under the influence of SPP1 alveolar macrophages; however, IL6 appears unrelated to other inflammatory and immune cells. Therefore, we suggest that tocilizumab (an anti-IL6-receptor antibody) be administered early in SSc-ILD to preserve lung function. We also observed a specific subset of macrophages that activate the MAPK signaling pathway. This could be the reason for the earlier onset and continuing fibrosis of SSc-ILD compared to IPF, primarily due to the higher occurrence of hypoxia and ROS pathways, and the absence of feedback inhibition of ANNEXIN from mast cells. We identified two TFs, BCLAF1 and NFE2L2, and their downstream target genes associated with MAPK, which may be potential therapeutic targets for early prevention and treatment. We found that metformin downregulated NFE2L2, which could serve as a repurposed drug candidate.

### Supplementary Information

Supplementary Material 1.

Supplementary Material 2.

Supplementary Material 3.

Supplementary Material 4.

Supplementary Material 5.

## Data Availability

The authors confirm that the data supporting the findings of this study are available in the article and its supplementary materials.

## Abbreviations

ILD: Interstitial lung disease
SSc: Systemic sclerosis
SSc-ILD: Systemic sclerosis-related interstitial lung disease
IPF: Idiopathic pulmonary fibrosis
GEO: Gene expression omnibus
scRNA-seq: Single-cell RNA-sequencing
HCs: Healthy controls
PCs: Principle components
SNN: Shared nearest neighbor
UMAP: Uniform manifold approximation and projection
DEGs: Differentially expressed genes
GO: Gene ontology
KEGG: Kyoto Encyclopedia of Genes and Genomes
TF: Transcription factor
SCENIC: Single-cell regulatory network inference and clustering
AUC: Area under the curve
NES: Normalized enrichment score
FDR: False discovery rate
MAPK: Mitogen-activated kinase phosphatase
EndMT: Endothelial-to-myofibroblast transition
RP: Raynaud phenomenon
DLCO: Diffusion capacity of carbon monoxide
ROS: Reactive oxygen species
HIF-1α: Hypoxia inducible factor-1α
