# Supplementary material for: Macrophages as determinants and regulators of systemic sclerosis-related interstitial lung disease
Source: J Transl Med. 2024 Jun 27;22:600. doi: 10.1186/s12967-024-05403-4 (PMC11212242; doi:10.1186/s12967-024-05403-4)

**Supplementary Fig. S1 Single-cell RNA-sequencing analysis of lung samples from sysetmic sclerosis (SSc), idiopathic pulmonary fibrosis (IPF) and healthy controls (HCs).**

1. A ranking of principle components based on the percentage of variance of “Elbow plot” in SSc-ILD.


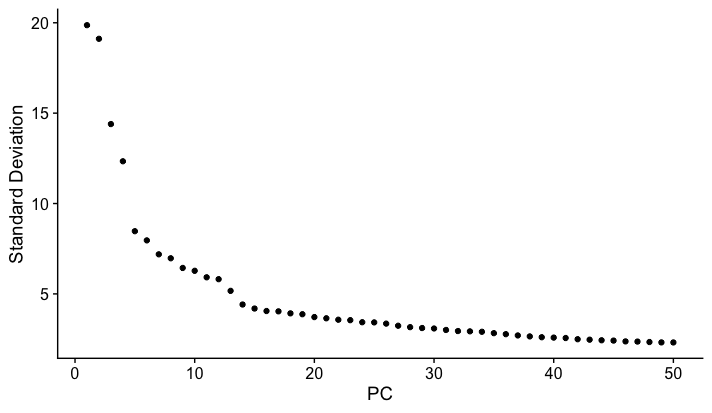


1. Partition of identity of cell clusters in SSc-ILD.


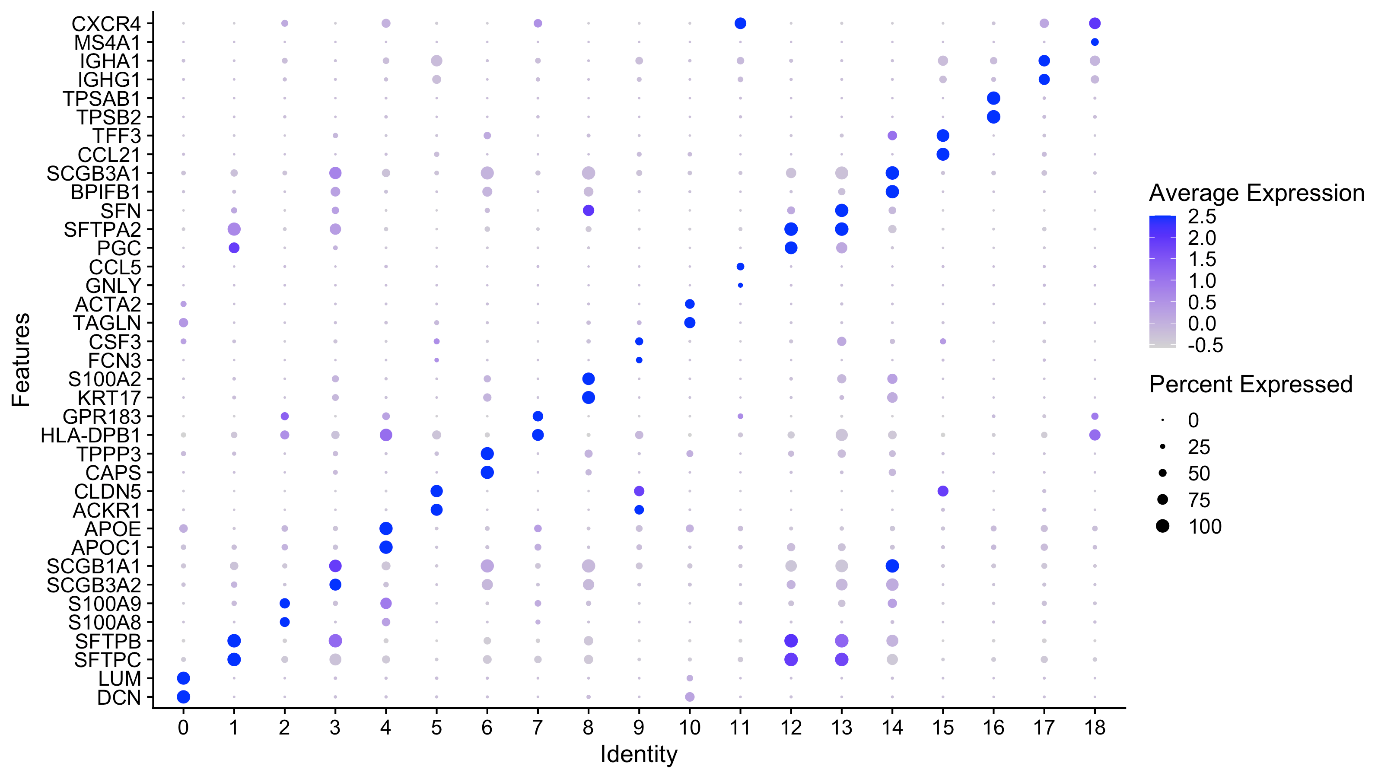


1. Differential expression of key genes violin plots in SSc-ILD of GSE159354. 0-fibroblast, 1-Type II alveolar cell, 2-monocyte, 3-secretory cell, 4-alveolar macrophage, 5-endothelial cell, 6-ciliated cell, 7-macrophage, 8-basal cell, 9-endothelial cell, 10-myofibroblast, 11-T/NK cell, 12-Type II alveolar cell, 13-epithelial cell, 14-club cell, 15-goblet cell, 16-mast cell, 17-B/plasma cell, 18-T/NK cell.


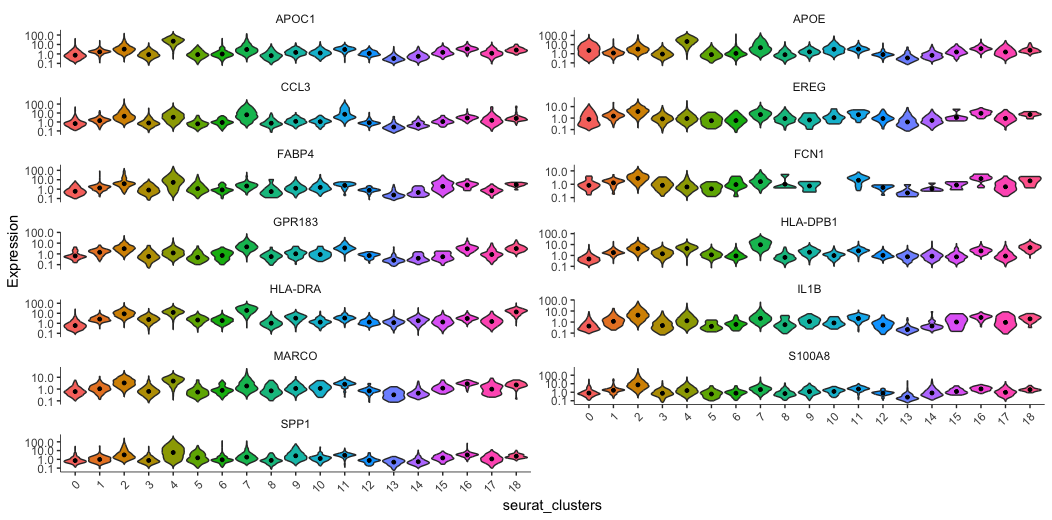


1. Gene ontology analysis of SSc-ILD.


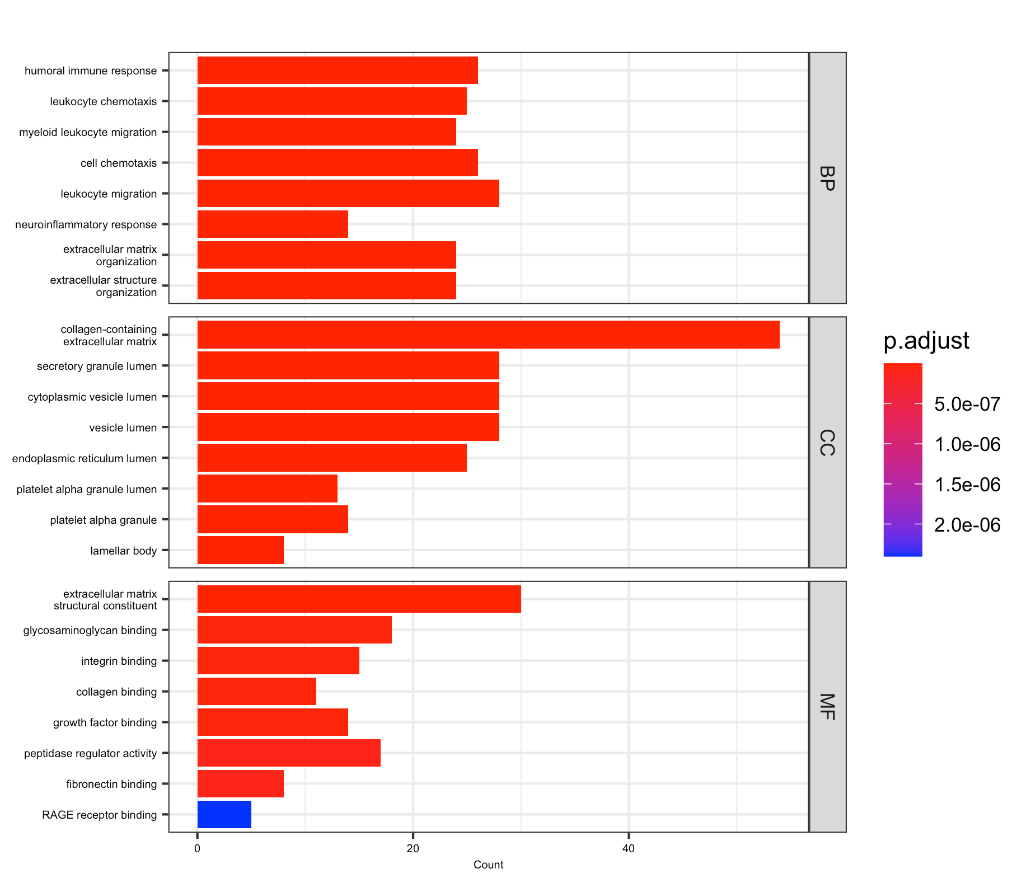


1. Gene ontology analysis of IPF.


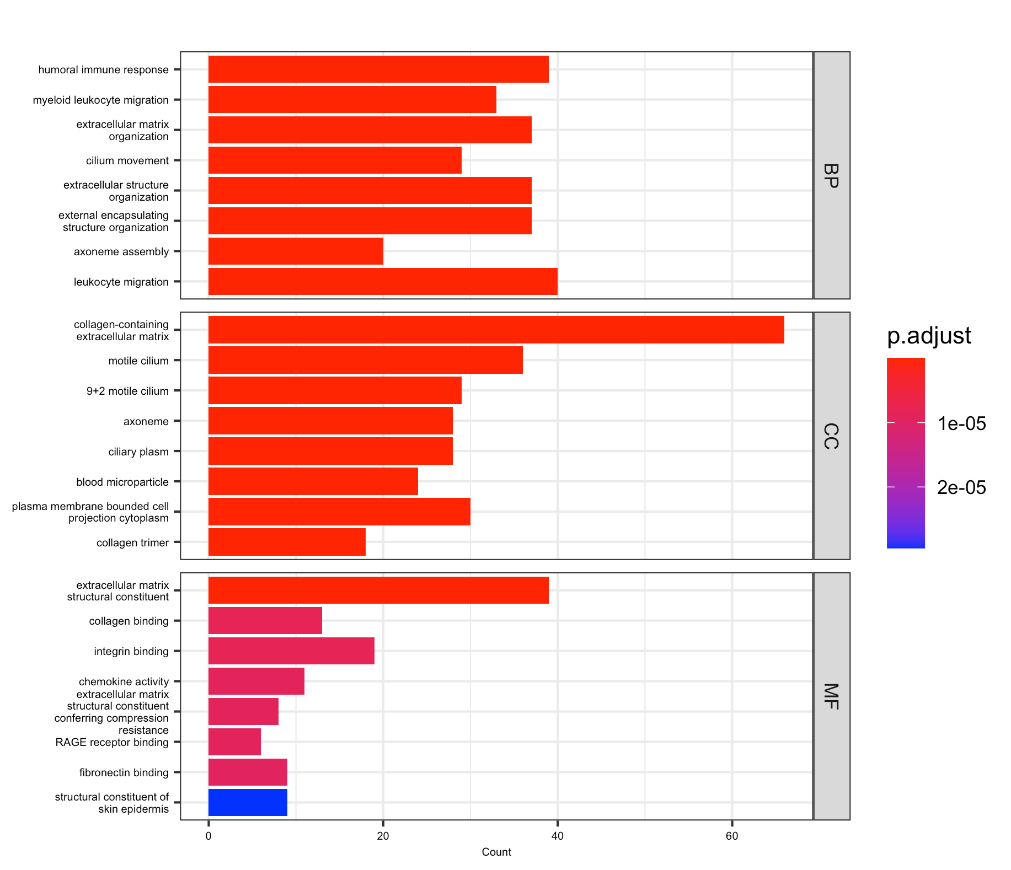


1. The MCODE analysis of SSc-ILD. The protein-protein interaction of MCODE_1 component of SSc-ILD and IPF.


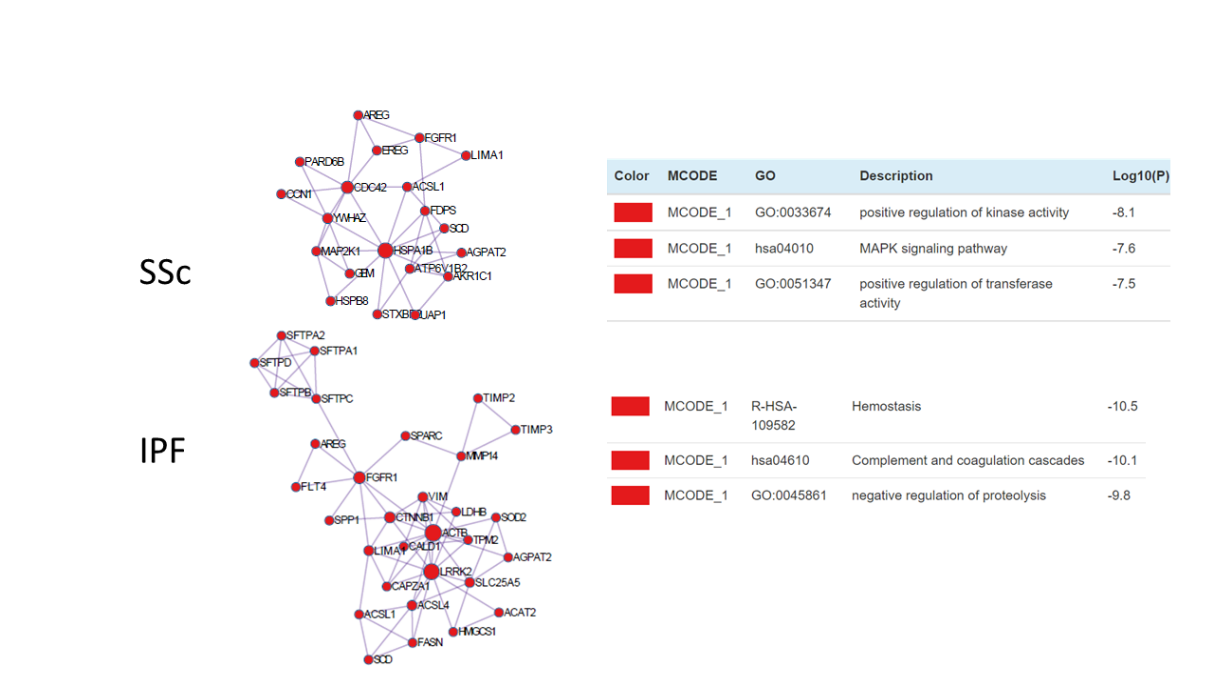


(G-J) KEGG pathway analysis of specific cell clusters. (G) Type II alveolar cell, (H) fibroblast, (I) alveolar macrophage, (J) monocyte.

| 1. Type II alveolar cell   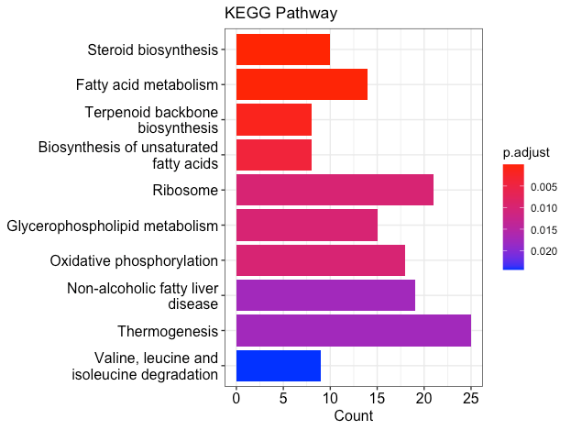 | 1. Fibroblast   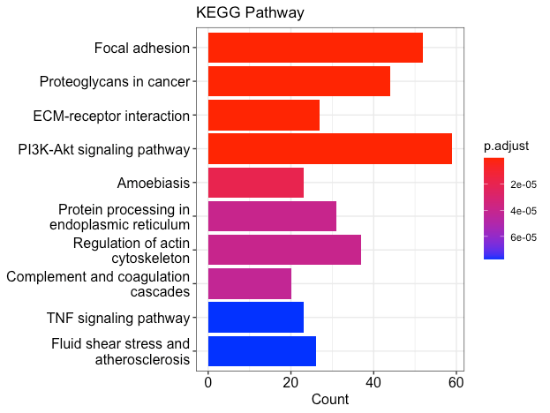 |
| --- | --- |
| 1. Alveolar Macrophage   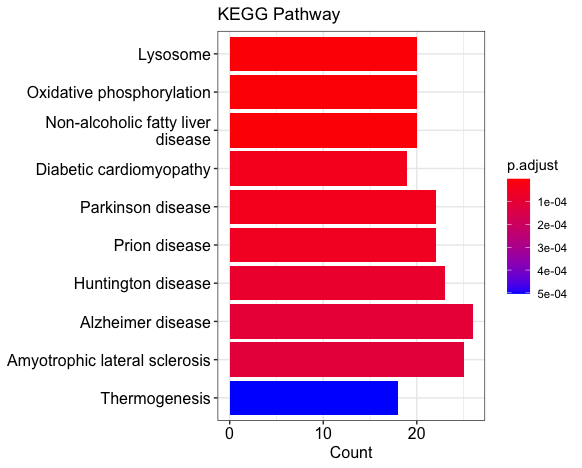 | 1. Monocyte   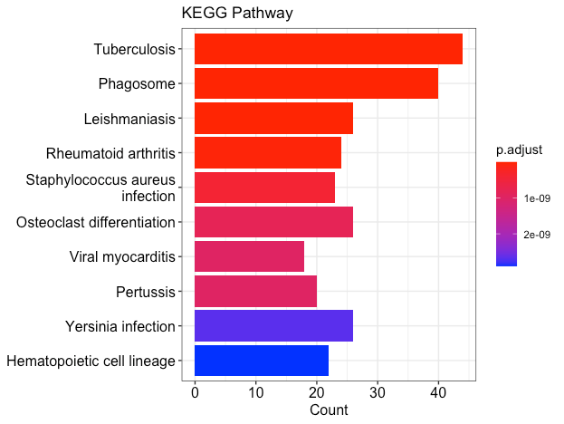 |

1. Suggested trajectory from monocyte, macrophage and alveolar macrophage of SSc-ILD on the 2D map.


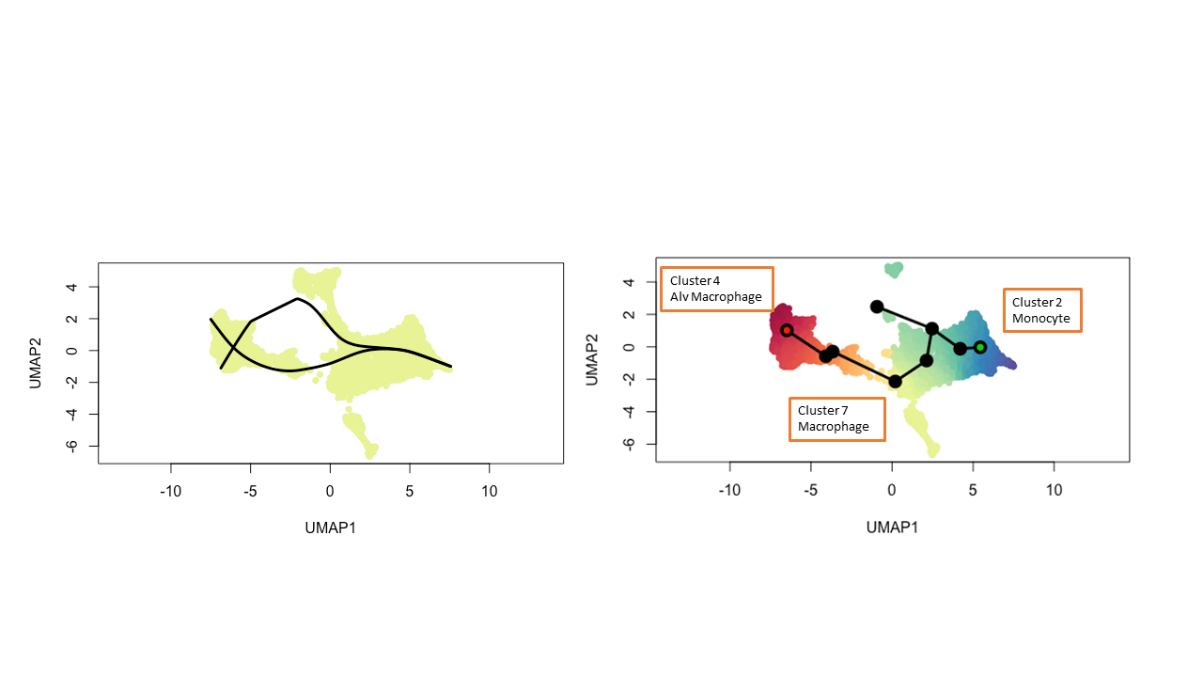


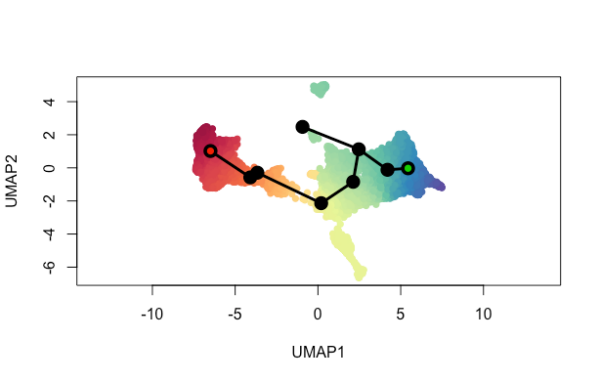

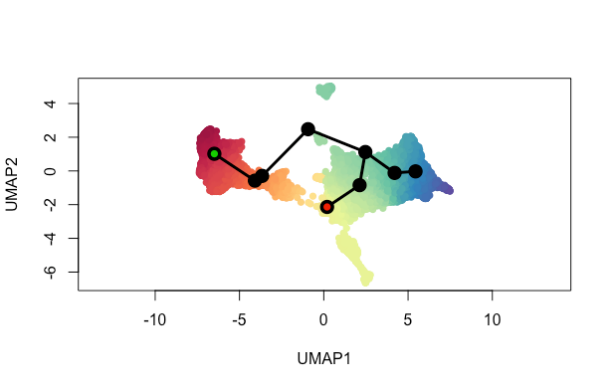


1. Differential expression of key genes violin plots in SSc-ILD of GSE212109. Clusters 2 and 7 correspond to monocytes, cluster 8 represents macrophages, and clusters 1, 3, 12, and 26 are alveolar macrophages.


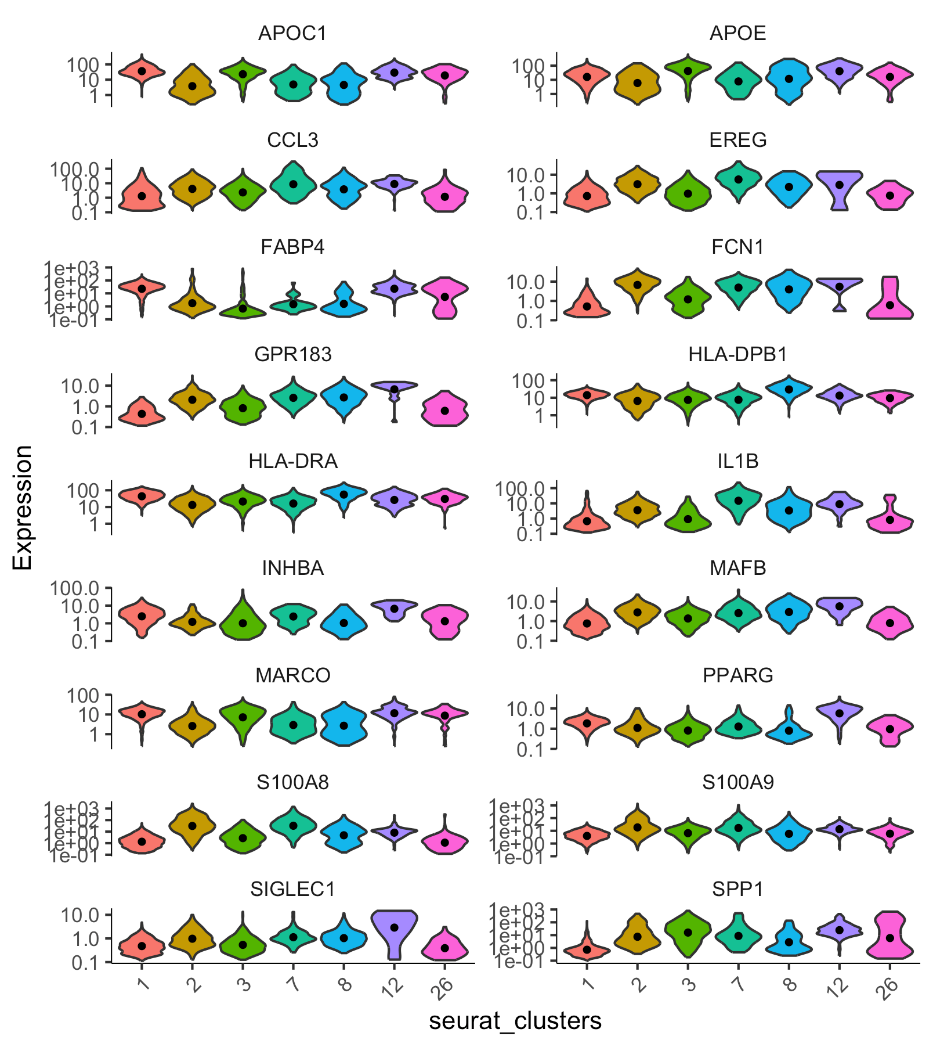

Supplement: Supplementary file 1 — Supplementary Material 1. [file 12967_2024_5403_MOESM1_ESM.docx]
