# Supplementary material for: Macrophages as determinants and regulators of systemic sclerosis-related interstitial lung disease
Source: J Transl Med. 2024 Jun 27;22:600. doi: 10.1186/s12967-024-05403-4 (PMC11212242; doi:10.1186/s12967-024-05403-4)

**Supplementary Fig.S2 Functional differences between cell cluster interactions in SSc and IPF. (A-F) Cell communication by ligand-receptor interaction in SSc-ILD. (A) fibroblast, (B) Type II alveolar cell, (C) alveolar macrophage, (D) monocyte, (E) macrophage, (F) ANNEXIN pathway of mast cell. (G) Cell communication by ligand-receptor interaction of mast cell in IPF.**

1. Fibroblast


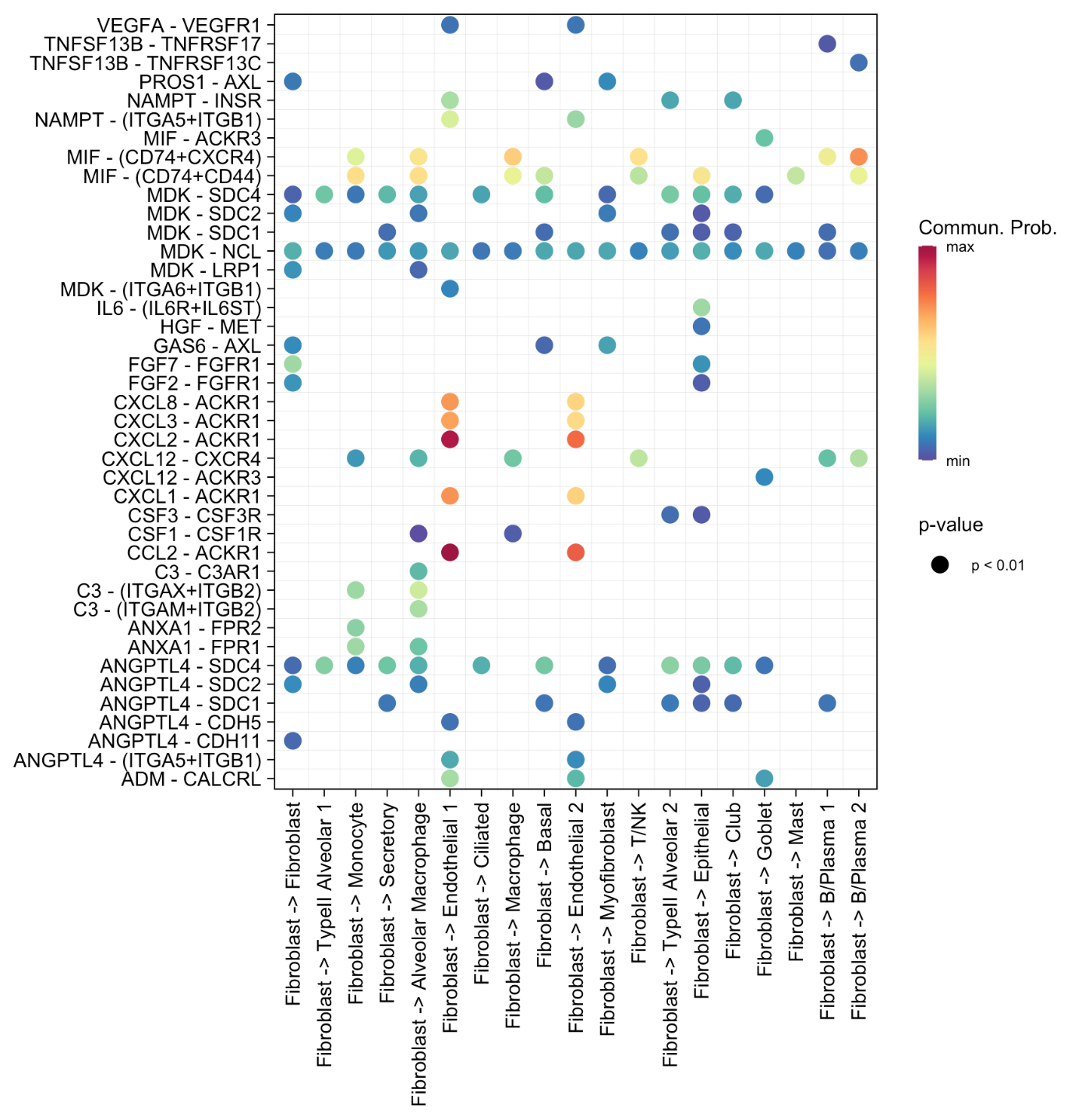


1. Type II Alveolar Cell


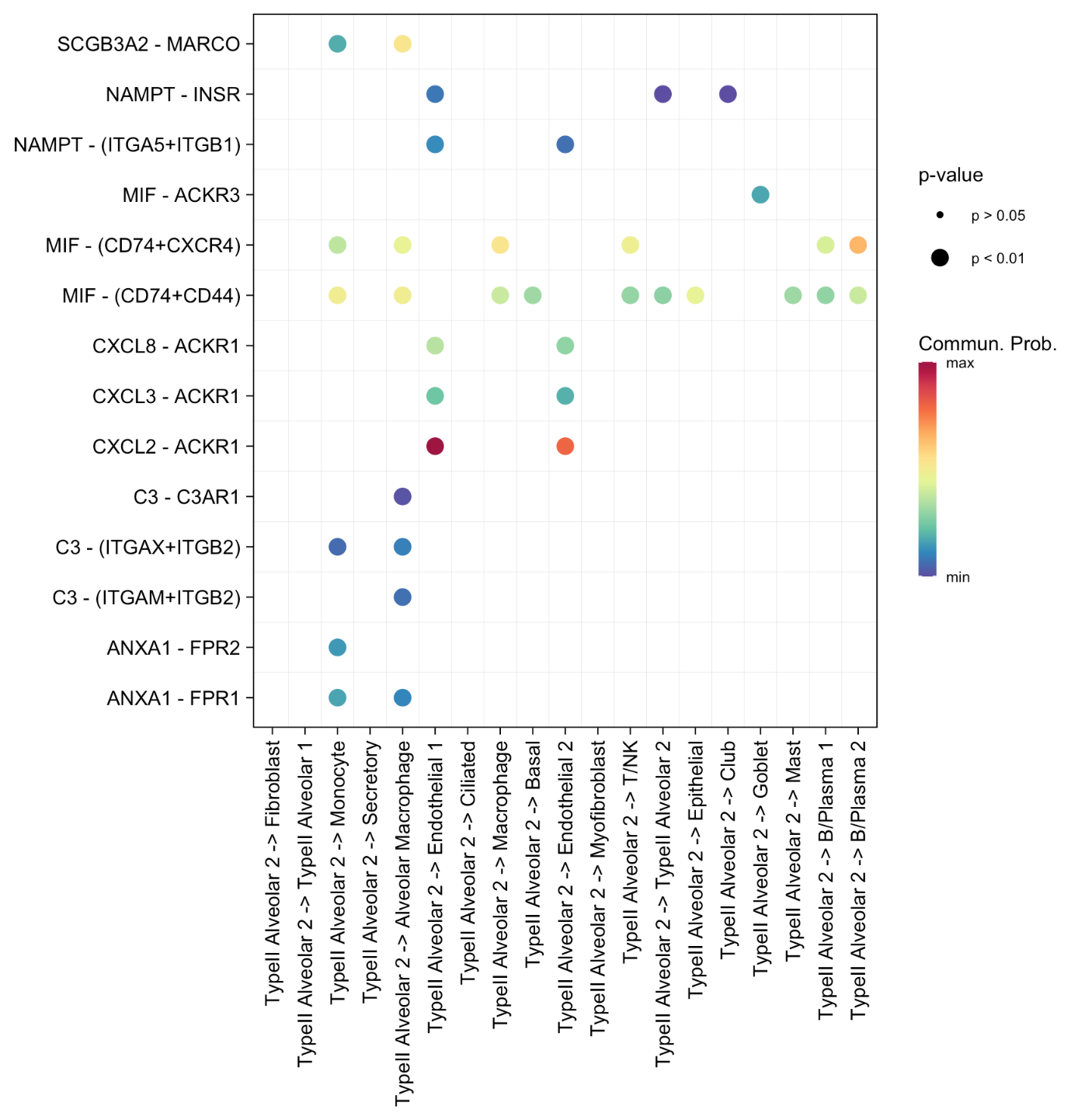


1. Alveolar Macrophage


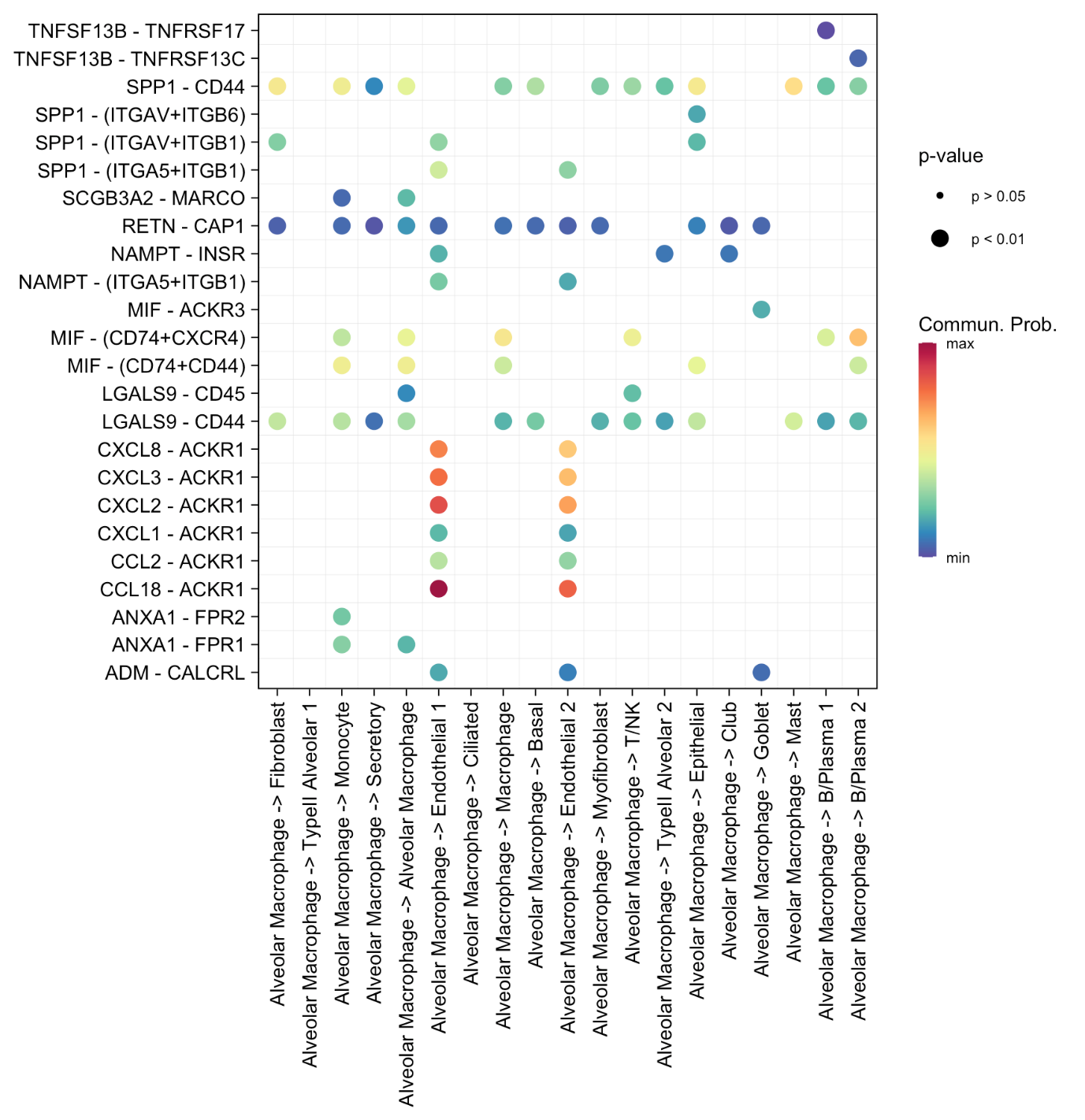


1. Monocyte


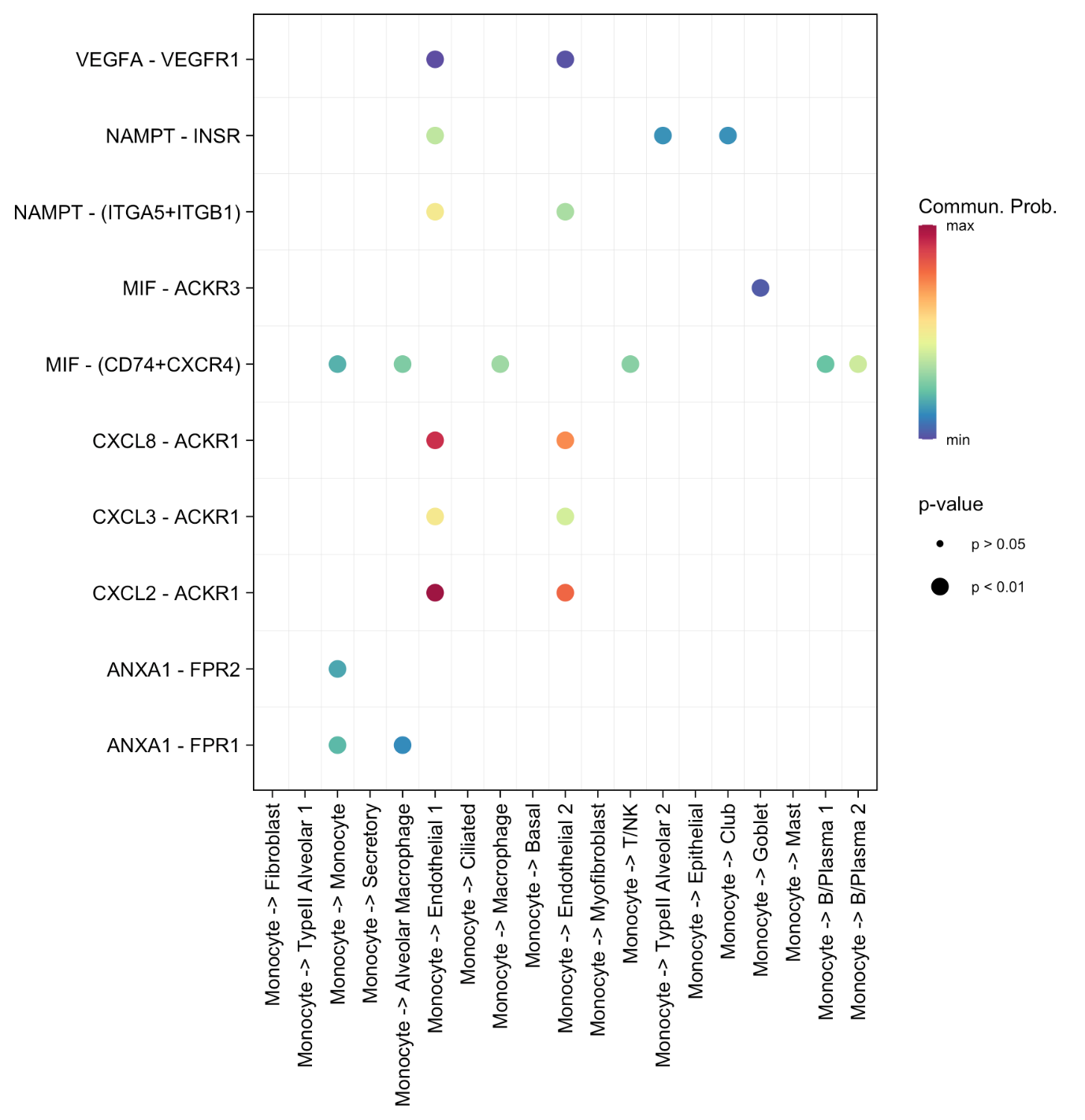


1. Macrophage


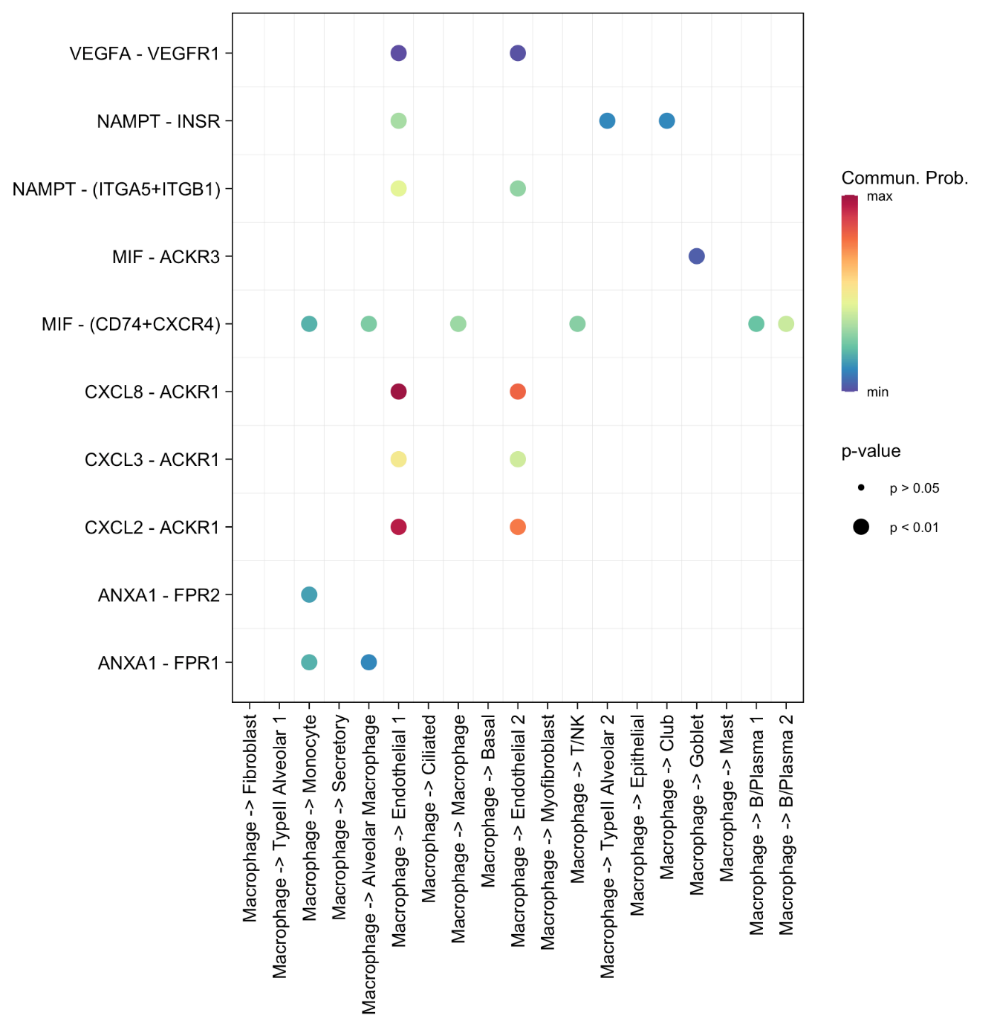


1. Mast cell in SSc-ILD


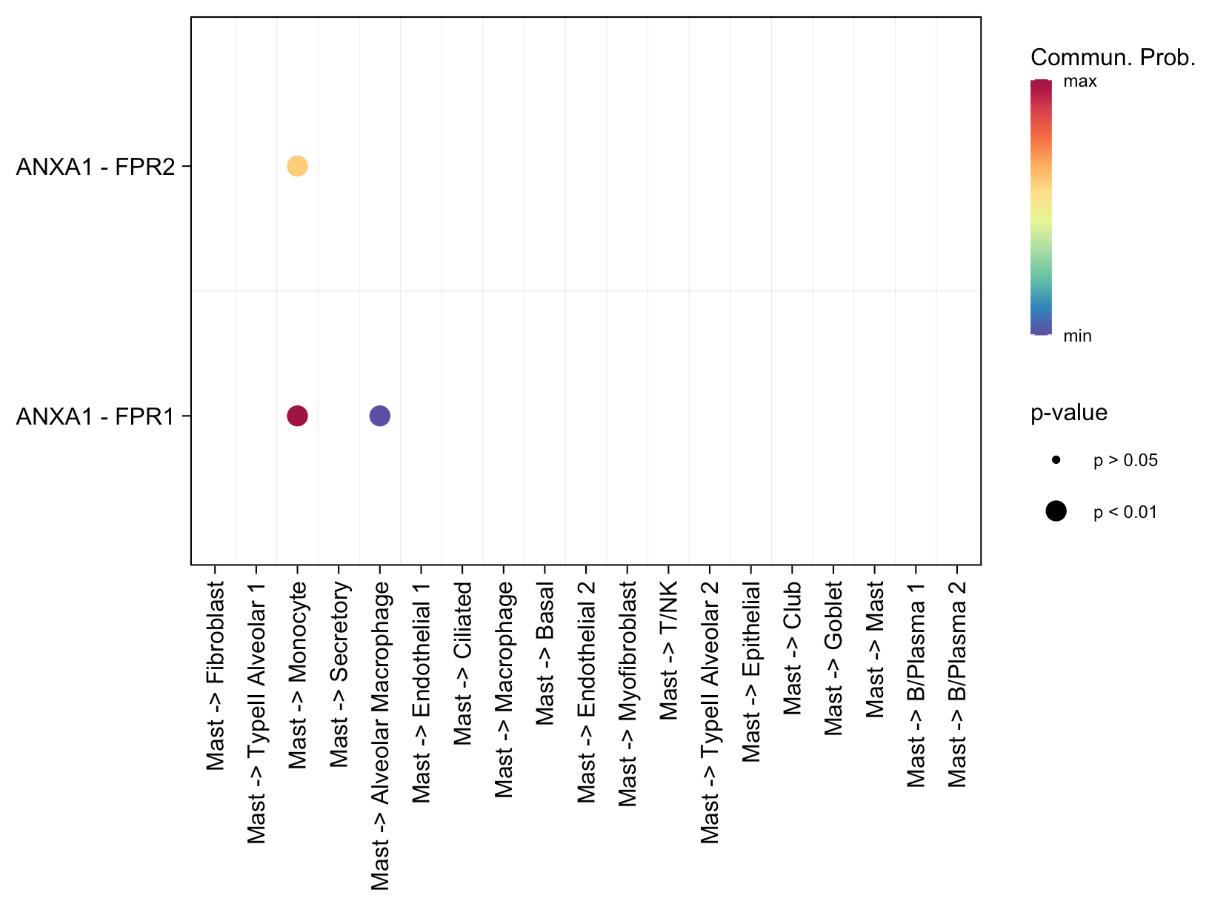


1. Mast cell in IPF


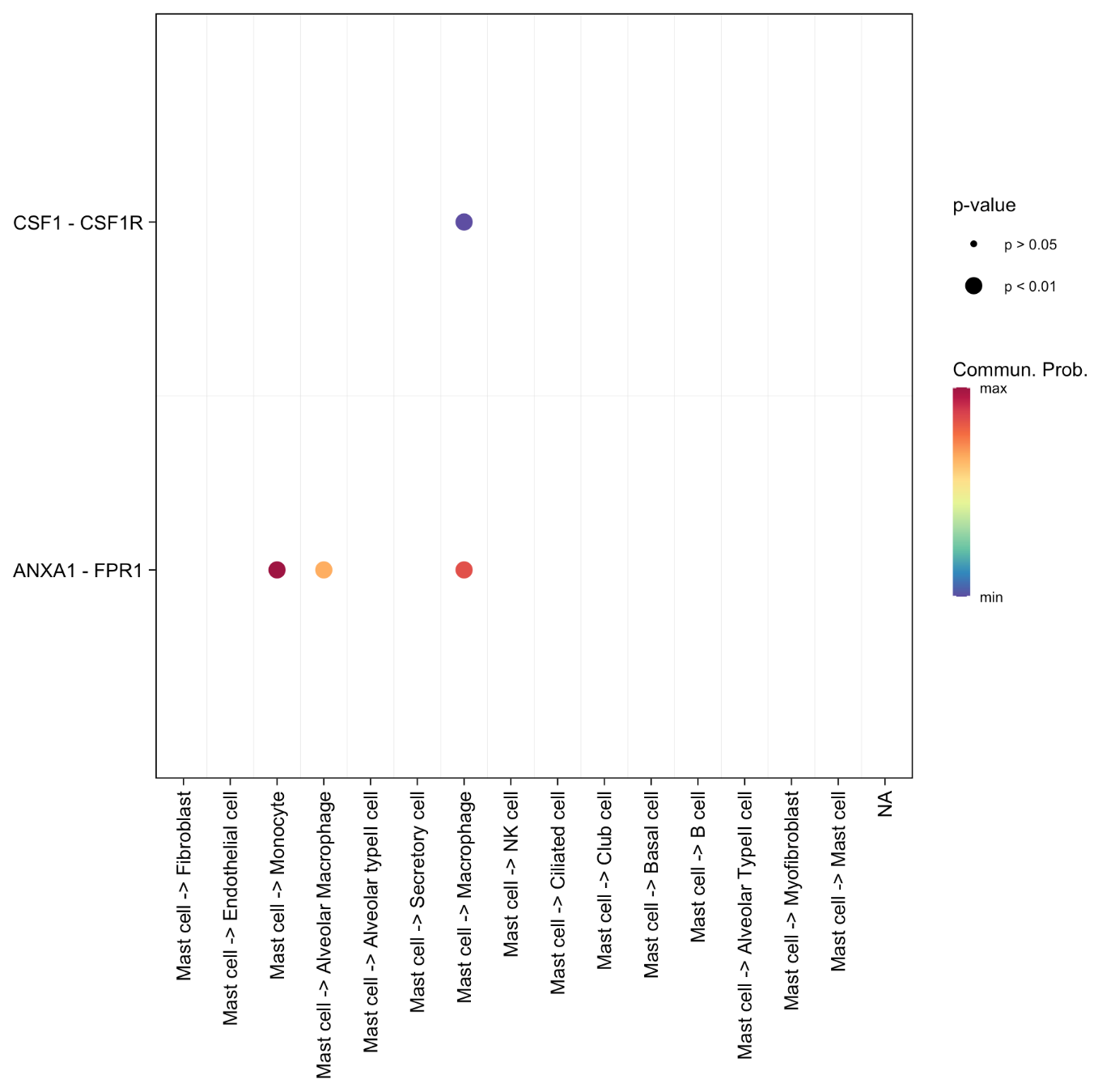

Supplement: Supplementary file 2 — Supplementary Material 2. [file 12967_2024_5403_MOESM2_ESM.docx]
