# Supplementary material for: Macrophages as determinants and regulators of systemic sclerosis-related interstitial lung disease
Source: J Transl Med. 2024 Jun 27;22:600. doi: 10.1186/s12967-024-05403-4 (PMC11212242; doi:10.1186/s12967-024-05403-4)
Supplement: Supplementary file 3 — Supplementary Material 3. [file 12967_2024_5403_MOESM3_ESM.docx]

**Supplementary Fig. S3 The regulon activity of tSNEs and AUC histograms. (A) BCLAF1, (B) NFE2L2, (C) JUN, (D) IRF1, (E) FOS, (F) FOSB.**


| 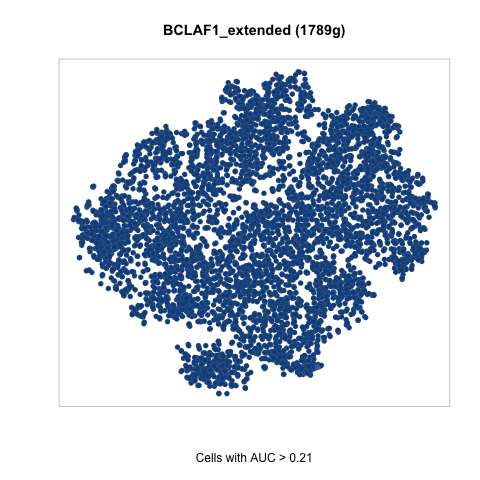 | 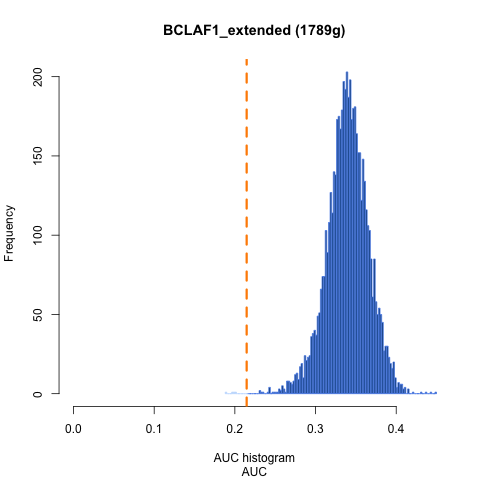 |
| --- | --- |


| 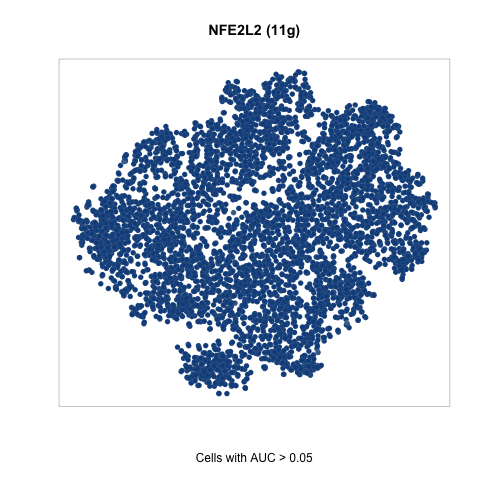 | 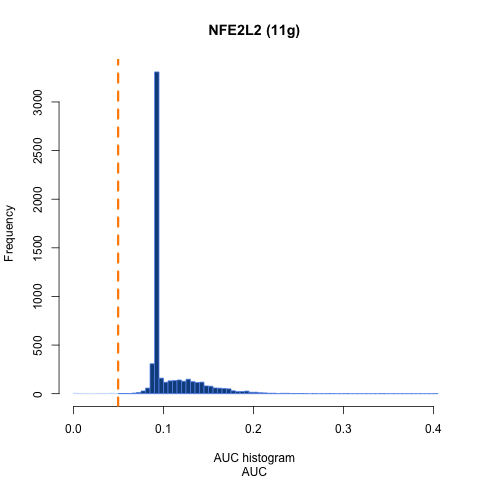 |
| --- | --- |


| 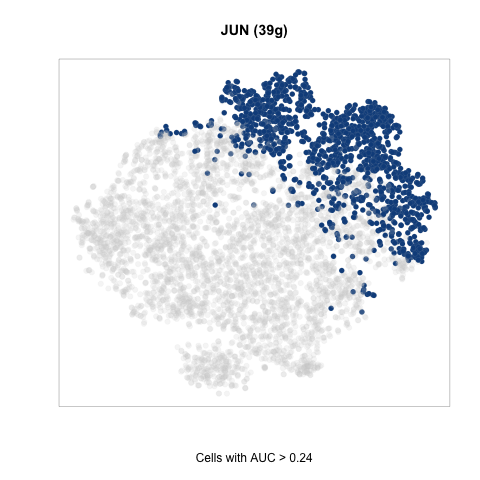 | 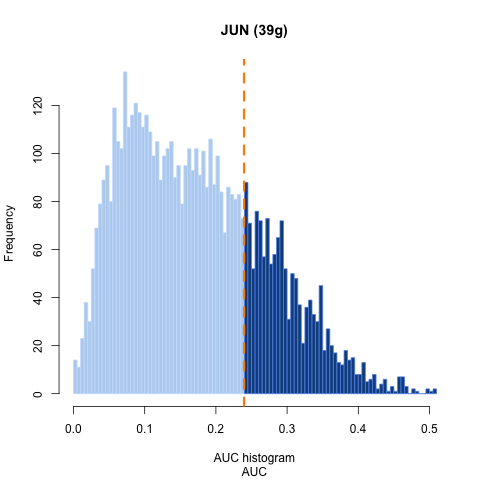 |
| --- | --- |


| 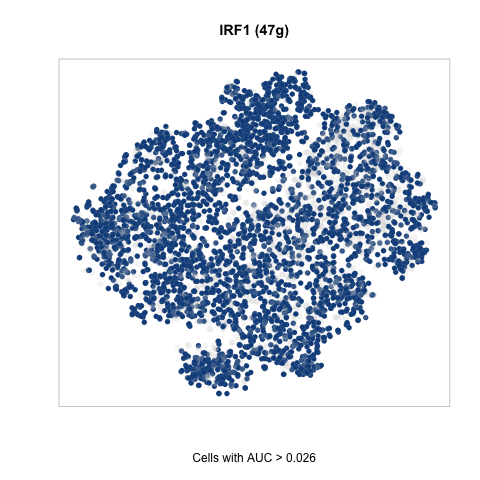 | 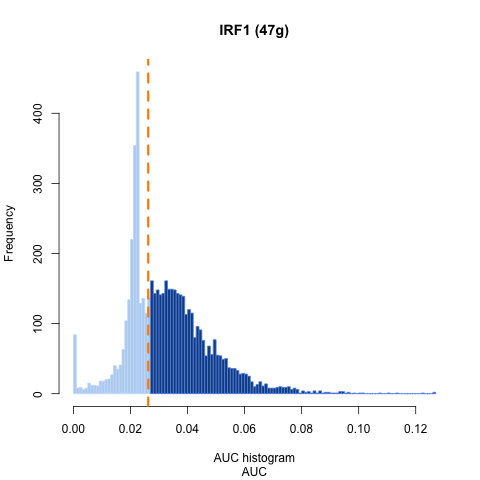 |
| --- | --- |


| 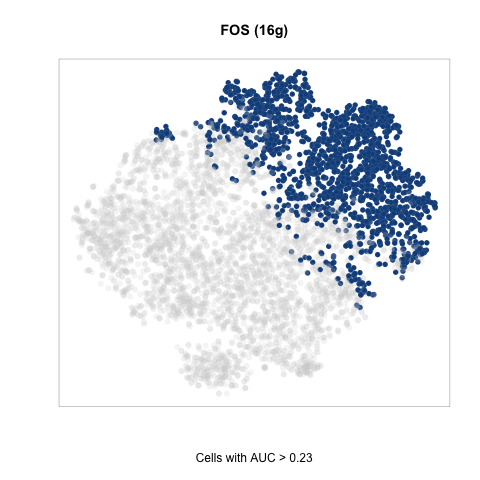 | 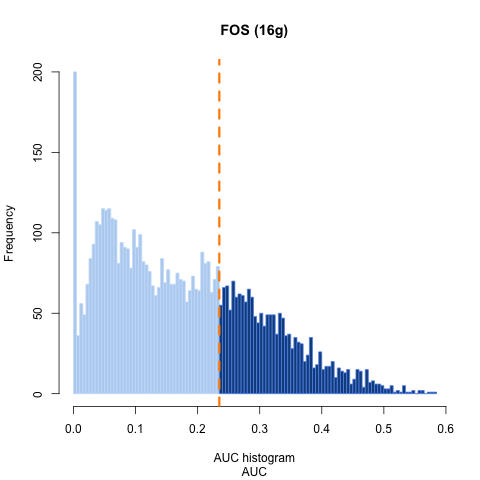 |
| --- | --- |


| 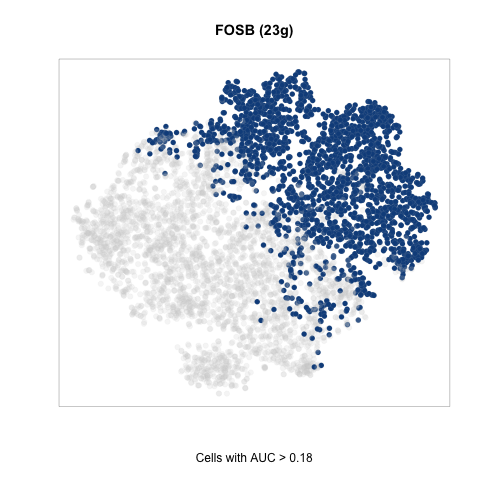 | 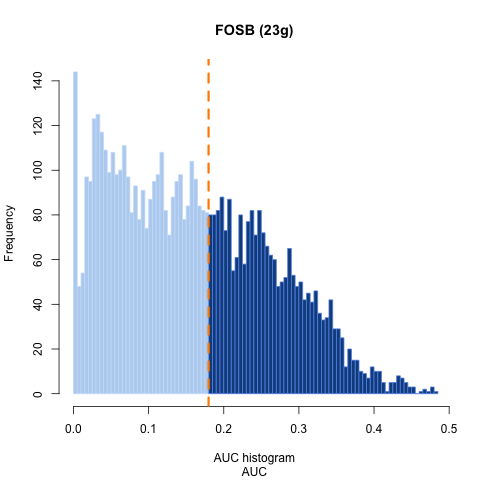 |
| --- | --- |
