# Supplementary material for: Macrophages as determinants and regulators of systemic sclerosis-related interstitial lung disease
Source: J Transl Med. 2024 Jun 27;22:600. doi: 10.1186/s12967-024-05403-4 (PMC11212242; doi:10.1186/s12967-024-05403-4)
Supplement: Supplementary file 4 — Supplementary Material 4. [file 12967_2024_5403_MOESM4_ESM.docx]

Table S1: The transcription factors with down-regulated genes in MAPK signaling pathway.

| TF | gene | highConfAnnot | nMotifs | bestMotif |  | NES | motifDb | coexModule | spearCor | CoexWeight |
| --- | --- | --- | --- | --- | --- | --- | --- | --- | --- | --- |
| BCLAF1 | MAP3K8 | FALSE | 1 | taipale_tf_pairs__ETS2_RCCGGAAGTG_HT |  | 4.79 | 10kb | w0.001 | 0.0745 | 0.0016 |
| BCLAF1 | TNF | FALSE | 2 | taipale_tf_pairs__ETS2_RCCGGAAGTG_HT |  | 4.79 | 10kb | w0.001 | 0.0808 | 0.0021 |
| BCLAF1 | MAP2K1 | FALSE | 1 | taipale_tf_pairs__ETS2_RCCGGAAGTG_HT |  | 3.14 | 500bp | w0.001 | 0.0923 | 0.0012 |
| BCLAF1 | MAP2K3 | FALSE | 1 | taipale_tf_pairs__ETS2_RCCGGAAGTG_HT |  | 3.14 | 500bp | w0.001 | 0.1118 | 0.0037 |
| BCLAF1 | NFE2L2 | FALSE | 1 | taipale_tf_pairs__ETS2_RCCGGAAGTG_HT |  | 4.79 | 10kb | w0.001 | 0.1143 | 0.0019 |
| BCLAF1 | DDIT3 | FALSE | 2 | taipale_tf_pairs__ETS2_RCCGGAAGTG_HT |  | 4.79 | 10kb | w0.001 | 0.0494 | 0.0014 |
| BCLAF1 | GADD45B | FALSE | 1 | taipale_tf_pairs__ETS2_RCCGGAAGTG_HT |  | 4.79 | 10kb | w0.001 | 0.0978 | 0.0016 |
| BCLAF1 | NR4A1 | FALSE | 2 | taipale_tf_pairs__ETS2_RCCGGAAGTG_HT |  | 4.79 | 10kb | w0.001 | 0.0960 | 0.0016 |
| BCLAF1 | DUSP2 | FALSE | 3 | taipale_tf_pairs__ETS2_RCCGGAAGTG_HT |  | 4.79 | 10kb | w0.001 | 0.0943 | 0.0014 |
| BCLAF1 | IRF1 | FALSE | 2 | taipale_tf_pairs__FOXO1_ELK3_RCCGGAWGTKKW_CAP |  | 3.67 | 10kb | w0.001 | 0.1139 | 0.0022 |
| NFE2L2 | MAP2K1 | FALSE | 1 | elemento__TGACTCA |  | 4.50 | 10kb | top3sd | 0.2548 | 0.0173 |
| NFE2L2 | MAP2K3 | FALSE | 1 | elemento__TGACTCA |  | 4.50 | 10kb | top3sd | 0.1738 | 0.0177 |
| NFE2L2 | IL1B | TRUE | 1 | transfac_pro__M02263 |  | 4.32 | 10kb | top50 | 0.2066 | 0.0205 |
| IRF1 | MAP3K8 | TRUE | 1 | tfdimers__MD00414 |  | 3.10 | 500bp | top1sd | 0.1891 | 0.0084 |
| IRF1 | TNF | TRUE | 2 | tfdimers__MD00527 |  | 3.22 | 500bp | top1sd | 0.1521 | 0.0077 |
| JUN | DDIT3 | TRUE | 4 | transfac_pro__M08939 |  | 4.31 | 10kb | top3sd | 0.3205 | 0.0498 |
| JUN | GADD45B | TRUE | 1 | transfac_pro__M08942 |  | 3.24 | 10kb | top3sd | 0.3438 | 0.0556 |
| JUN | NR4A1 | TRUE | 4 | transfac_pro__M08937 |  | 4.90 | 10kb | top3sd | 0.3941 | 0.0461 |
| JUN | DUSP2 | TRUE | 5 | transfac_pro__M08937 |  | 4.90 | 10kb | top3sd | 0.3635 | 0.0372 |
| FOS | JUN | TRUE | 3 | transfac_pro__M08943 |  | 4.07 | 10kb | top50 | 0.6462 | 0.1579 |
| FOS | NR4A1 | TRUE | 2 | transfac_pro__M07666 |  | 3.42 | 10kb | top50 | 0.3878 | 0.0631 |
| FOSB | NR4A1 | TRUE | 2 | transfac_pro__M08921 |  | 3.77 | 10kb | top3sd | 0.3311 | 0.0310 |
| FOSB | DUSP2 | FALSE | 1 | scertf_badis.CST6 |  | 4.12 | 10kb | top3sd | 0.2878 | 0.0238 |

TF: transcription factor

coexModule: co-expression module

　w0.001: In each TF, retain genes with weight > 0.001 to form modules.

　top1sd: Retain targets in each TF where the weight is greater than mean(weight) + sd(weight).

　top3sd: Retain targets in each TF where the weight is greater than mean(weight) + 3*sd(weight).

　top50: Retain the top 50 TFs based on weight for each gene to obtain a simplified TF-target pairs list. Then, assign genes to TFs to construct co-expression modules.
