# Supplementary material for: Macrophages as determinants and regulators of systemic sclerosis-related interstitial lung disease
Source: J Transl Med. 2024 Jun 27;22:600. doi: 10.1186/s12967-024-05403-4 (PMC11212242; doi:10.1186/s12967-024-05403-4)
Supplement: Supplementary file 5 — Supplementary Material 5. [file 12967_2024_5403_MOESM5_ESM.docx]

Table S2: The up-regulated and down-regulated effects of drugs to specific transcription factors.

|  | Up-regulated | Down-regulated | |
| --- | --- | --- | --- |
| TF |  | Candidate | Controversial |
| BCLAF1 | Acetaminophen, Capsaicin, Thimerosal, | Tretinoin,  Belinostat, Vorinostat | DinitrochlorobenzeneEugenol, Formaldehyde, Selenium, Tamibarotene, Valproate, Vincristine,  Vit E |
| NFE2L2 | Acetaminophen, Cyclosporin, Calcitriol, Icosapent, Ellagic acid, Ginsenoside (Rb1, Rg1), Heparin, Hydrogen peroxide, Ibuprofen, Medroxyprogesterone acetate, Naringenin, Panobinostat, Resveratrol, Seocalcitol, Sertraline, Silver, Lipoic acid, Torcetrapib, Zoledronic acid | Metformin | Estradiol, Oxygen, Quercetin, Selenium, Silver nitrate, Tamibarotene, Tretinoin, Valproate,  Vit E |
| IRF1 | Alitretinoin, Amiodarone, Acetylsalicylic acid, Cyclosporin, Dacarbazine, Decitabine, Dinitrochlorobenzene, Formaldehyde, Fenretinide, Methotrexate, Silicon dioxide, Tamibarotene, Titanium dioxide, Tretinoin, Troglitazone, Vincristine |  | Arsenic trioxide, Calcitriol,  Silver |
| JUN | Acetaminophen, Aluminum oxide, Anisomycin, Arachidonic acid, Indomethacin, Acetylsalicylic acid, Azathioprine, Copper, Cupric chloride, Curcumin, Cytarabine, Deferoxamine, Dronabinol, Cisplatin, Dinitrochlorobenzene, Fenthion, Fluorouracil, Formaldehyde, Fotemustine, Fulvestrant, Genistein, Hemin, Irinotecan, iron, Mitoxantrone, Nefazodone, Oxygen, Panobinostat, Quercetin, Raloxifene, Sertraline, Silicon dioxide, Silver, Sorafenib, Thalidomide, Thimerosal, Torcetrapib, Valproic acid, Vincristine, Vitamin D, Zinc | Masoprocol | Arbutin, Arsenic trioxide, Estradiol, Bortezomib, Capsaicin, Cyclosporin, Ellagic acid, Fluoxetine, Hydrogen peroxide, Lycopene, Resveratrol, Vitamin E, Vorinostat |
| FOS | Acebutolol, Acetaminophen, Acrivastine, Afimoxefene, Allopurinol, Stanolone, Amiloride, Amiodarone, Anisomycin, Arachidonic acid, Azathioprine, Bazedoxifene, Benzofenone, Calyculin A, Chlorpopamide, Chromium, Cupric chloride, Cupric oxide, Cyclosporin, Daidzin, Dronabinol, Dexamethasone, Diclofenac, Diflunisal, Calcitriol, Dinitrochlorobenzene, Diphencyprone, Dipyridamole, Ephedrine, Ethinylestradiol, Fenthion, Flecainide, Fluorouracil, Formaldehyde, Fotemustine, Genistein, Ginsenoside Rg1, Hemin, Histamine, Lithium cation, Lycopen, Mebendazole, Methylphenidate, Mycophenolic acid, Nabumetone, Nefazodone, Nicotine, Nimodipone, Oxygen, Pentoxifylline, Dinoprostone, Protoporphyrin, Puerarin, Pyrimethamine, Quercetin, Reservatrol, Sertraline, Silver, Silver nitrate, Sodium chloride, Selenium, Tamoxifen, Thalidomide, Thimerosal, Torcetrapib, Trinitrotoluene, Valproic acid, Verapamil, Vitamin D, Zinc sulfate, | Disulfiram | Aluminum oxide, Arsenic trioxide, Acetylsalicylic acid, Estradiol, Capsaicin, Copper, Curcumin, Doxorubicin, Entinostat, Fluoxetine, Fulvestrant, Hydrogen peroxide, Hydroquinone, Irinotecan, Oxyquinoline, Silicon dioxide, Simvastatin, Testosterone, Tretinoin, Troglitazone, Zoledronic acid |
| FOSB | Tretinoin, Valproate, Anisomycin, Amiodarone, Acetaminophen, Estradiol, Cupric oxide, Dronabinol, Dexamethasone, Formaldehyde, Ibuprofen, Irinotecan, Lithium cation, Melphalan, Nicotine, Silicon dioxide, Tamibarotene, Thalidomide, Thimerosal, Tretinoin, Valproic acid | Disulfiram | Acetylsalicylic acid, Copper, Isotretinoin, Silver, Silver nitrate, |

TF: transcription factor
